# Risk factors for urinary tract infection in geriatric hip fracture patients: a systematic review and meta-analysis

**DOI:** 10.3389/fmed.2024.1360058

**Published:** 2024-02-09

**Authors:** Wei Wang, Wei Yao, Wanyun Tang, Yuhao Li, Hongbo Sun, Wenbo Ding

**Affiliations:** Department of Orthopedics, Dandong Central Hospital, China Medical University, Dandong, China

**Keywords:** urinary tract infection, UTI, hip fracture, risk factors, meta-analysis

## Abstract

**Background:**

Urinary tract infection (UTI) is a prevalent and consequential complication in hip fracture patients, leading to significant disability and heightened healthcare expenditures. Consequently, there is a critical need for a comprehensive systematic review to identify risk factors and establish early and effective preventive measures.

**Methods:**

A comprehensive search was performed across the PubMed, Cochrane, Embase, Web of Science, and Scopus databases (up to August 31, 2023). Article screening, data extraction, and quality assessment were independently completed by two reviewers.

**Results:**

Forty-four studies were eligible for inclusion, yielding an overall incidence rate of 11% (95% CI: 8%−14%). Our pooled analysis revealed 18 significant risk factors, including being female (OR = 2.23, 95% CI: 1.89–2.63), advanced age (MD = 1.35, 95% CI: 0.04–2.66), obesity (OR = 1.21, 95% CI: 1.11–1.31), catheterization (OR = 3.8, 95% CI: 2.29–6.32), blood transfusion (OR = 1.39, 95% CI: 1.21–1.58), American Society of Anesthesiologists ≥III (OR = 1.28, 95% CI: 1.18–1.40), general anesthesia (OR = 1.26, 95% CI: 1.11–1.43), intertrochanteric fracture (OR = 1.25, 95% CI: 1.01–1.54), hemiarthroplasty (OR = 1.43, 95% CI: 1.19–1.69), prolonged length of hospital stay (MD = 1.44, 95% CI: 0.66–2.23), delirium (OR = 2.66, 95% CI: 2.05–3.47), dementia (OR = 1.82, 95% CI: 1.62–2.06), Parkinson's disease (OR = 1.53, 95% CI: 1.46–1.61), diabetes (OR = 1.27, 95% CI: 1.13–1.43), hypertension (OR = 1.14, 95% CI: 1.03–1.26), congestive heart failure (OR = 1.35, 95% CI: 1.10–1.66), history of sepsis (OR = 7.13, 95% CI: 5.51–9.22), and chronic steroid use (OR = 1.29, 95% CI: 1.06–1.57).

**Conclusion:**

Our study identifies numerous risk factors strongly associated with UTI, offering compelling evidence and actionable strategies for improving clinical prediction, enabling early intervention, and facilitating targeted UTI management.

**Systematic review registration:**

identifier [CRD42023459600], https://www.crd.york.ac.uk/PROSPERO/display_record.php?RecordID=459600.

## Introduction

Hip fracture, the prevailing type of fracture in the geriatric populace, has garnered substantial attention globally. Reports indicate an alarming annual global incidence of approximately 1.6 million cases, accompanied by an exorbitant sum surpassing 10 billion US dollars in medical expenses (1–3). Moreover, owing to the progressive aging of populations, it is anticipated that hip fracture occurrences will experience a rapid annual increase ranging from 1% to 3% (4). This trend forecasts a staggering projection of approximately 6.1 million global hip fracture cases by the year 2050. Unfortunately, the elderly population afflicted with hip fractures is plagued by a multitude of pre-existing conditions, physical deterioration, and prolonged immobilization, thus yielding a complication rate ranging from 7% to 40% (5). Among these complications, UTI stands out as a prominent affliction afflicting hip fracture patients, with occurrence rates ranging from 4% to 32% (6, 7). The grave implications of UTIs are correlated with elevated mortality rates, profound disabilities, and escalated healthcare expenditures, ultimately dealing an irrevocable blow to the already vulnerable elderly hip fracture population.

To date, numerous systematic reviews have reported on other associated complications of hip fractures, such as pneumonia (3, 8), delirium (9), and deep vein thrombosis (10). However, no systematic review has been conducted to identify potential risk factors for UTI in hip fracture patients. Therefore, identifying the risk factors for UTI in hip fracture patients is crucial for clinical physicians to identify high-risk patients, guide targeted early interventions, and predict patient outcomes. This systematic review aims to address two crucial questions: (1) What is the incidence rate of UTI in hip fracture patients? (2) What are the related risk factors for UTI in hip fracture patients?

## Methods

This study has been registered on PROSPERO (CRD42023459600) and conducted according to the guidelines of Meta-analysis Of Observational Studies in Epidemiology (MOOSE).

### Search strategy

A comprehensive search was performed on the PubMed, Cochrane, Embase, Web of Science, and Scopus databases from their inception to August 31, 2023, to identify all relevant studies. To mitigate the inclusion of irrelevant articles, Keywords and relevant terms, such as “hip fracture,” “urinary tract infection,” and “risk factors,” were concatenated using the Boolean operator “AND.” The search was conducted without language or country restrictions. Furthermore, to prevent the omission of relevant primary studies, we manually reviewed the references cited in the primary studies and reviews.

### Study selection

The inclusion criteria for studies are as follows: (1) cohort or case-control studies; (2) restricted to patients with hip fractures, but excluding cases caused by multiple traumas; (3) The studies should report risk factors associated with UTI in patients with hip fractures that have been documented at least twice; (4) The diagnostic criteria for UTI are well-defined, and the occurrence of UTI is observed during the patient's hospitalization. The following will be excluded: (1) letters, comments, case reports, conference records, and animal studies; (2) inability to obtain full text, data duplication, or inability to calculate odds ratios (OR), mean difference (MD), and 95% confidence intervals (CI). The review will be excluded after manually reviewing the references.

A summary of all relevant studies retrieved, excluding duplicate records, was conducted for screening. To ensure the objectivity of the review results, the titles and abstracts of all articles were independently examined by two reviewers (WW and WY). Studies that met the criteria underwent an independent full-text review by the same reviewers, resulting in the final inclusion of studies. After each round of screening, the results were compared, and any discrepancies were thoroughly discussed to reach a consensus. In cases where consensus could not be reached, a third-party reviewer (WBD) was consulted for resolution.

### Data extraction

A standardized electronic form was utilized to extract the following data from the included studies: author, year of publication, country, study type, sample size, number of UTI patients, UTI diagnostic criteria, and relevant risk factors. The entire process was independently completed by two reviewers (WW and WY) who compared the extracted data. Comprehensive discussion was used to resolve any discrepancies, and in cases where a consensus could not be reached, a third-party reviewer (WBD) was consulted for resolution.

### Quality assessment

The quality of the included studies was independently assessed by two reviewers (WW and WY) using the Newcastle-Ottawa Quality Assessment Scale (NOS). The NOS is a tool used for systematically evaluating the quality of non-randomized controlled studies. It consists of 3 dimensions (selection, comparability, and outcome or exposure) and 8 items. One point is awarded for each fulfilled requirement, with a total score ranging from 0 to 9 (11). Only studies that meet the majority of the requirements (≥6 points) are considered to be of high quality. Any disagreements in scoring were resolved through discussion or consultation with a third-party reviewer (WBD).

### Statistical analysis

To obtain the pooled incidence rate, a meta-analysis was performed using the inverse variance method and random effects model in STATA 15.0 (STATA Corporation, College Station, TX, USA). Heterogeneity was assessed by I^2^ and chi-squared tests. If heterogeneity was present (I^2^ > 50% or *P* < 0.1), a meta-regression was used to explore the sources of heterogeneity.

When more than two studies reported the same risk factor, a meta-analysis was conducted using RevMan 5.4 (The Cochrane Collaboration, Oxford, UK). A random effects model was pre-specified, and the inverse variance or Mantel-Haenszel methods were used to estimate the pooled OR or MD depending on the data type of the risk factor (continuous or dichotomous). The effect model was adjusted according to the heterogeneity of the results. When significant heterogeneity was observed among studies (I^2^ > 50% or *P* < 0.1), a random effects model was used; otherwise, a fixed effects model was applied.

Sensitivity analyses were conducted to test the reliability and stability of the results by repeatedly excluding individual studies and examining changes in the pooled effect. Publication bias was assessed using funnel plots, Begg's test, and Egger's test. If publication bias was detected, the trim-and-fill method was used to adjust for it. These parts of analyses were conducted in STATA 15.0.

A two-tailed *P* value < 0.05 was considered statistically significant. There were no unavailable effect sizes or 95% CIs in our study.

## Results

### Study selection and quality assessment

**Figure 1:**
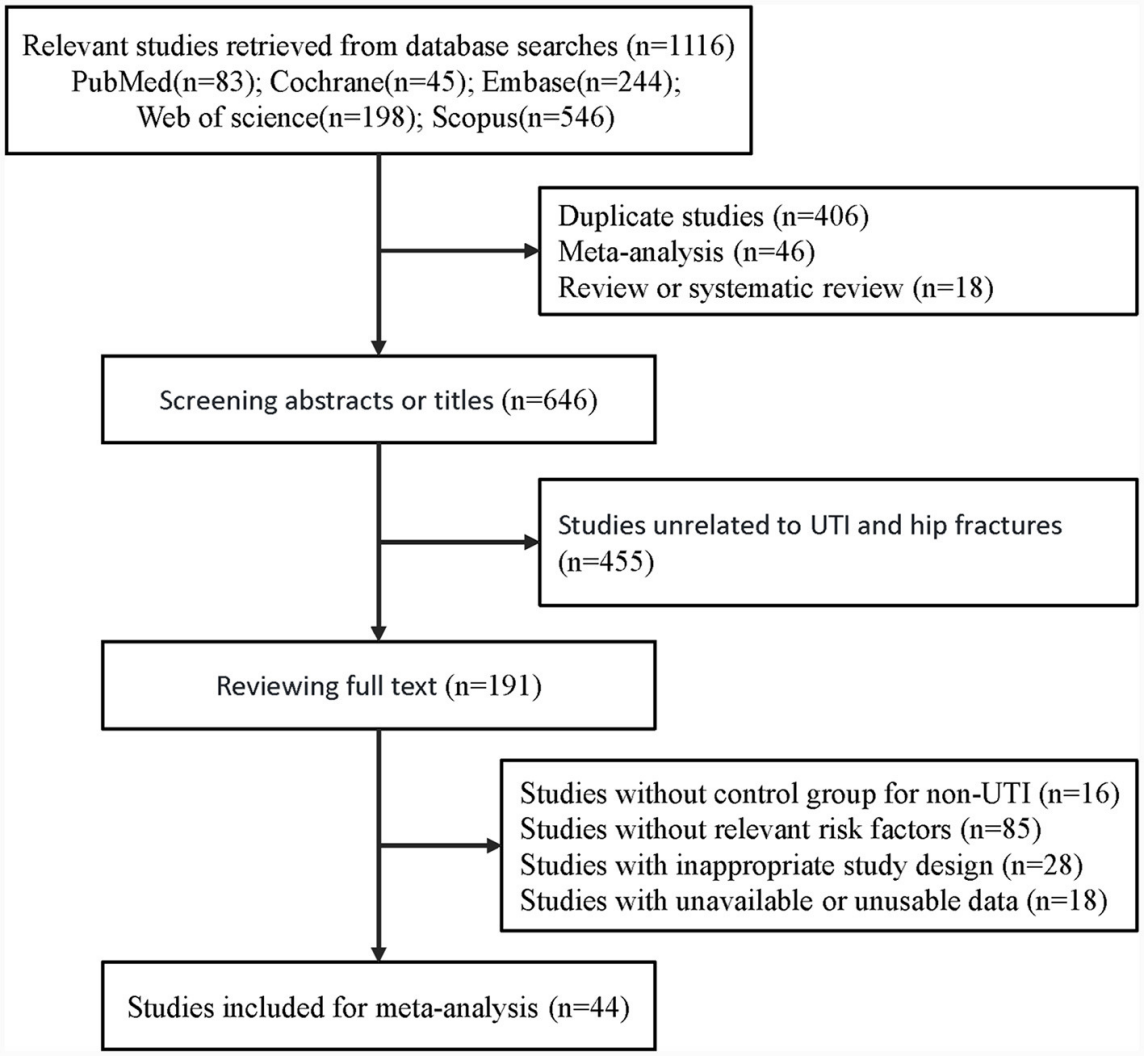
Flowchart of study selection.

A total of 1,116 articles were initially collected through searching five databases (all articles obtained from manual review of reference lists were included in the articles retrieved from the database search). After excluding duplicates and reviews, the titles and abstracts of 646 articles were screened, resulting in the removal of 455 articles that did not align with our research topic. Full-text review was performed on the remaining 191 articles. Ultimately, 44 studies that met our inclusion criteria were included in this meta-analysis. All included studies were English articles, primarily sourced from Europe, the Americas, and Asia. A detailed flowchart of study selection process is presented in Figure 1.

The quality of non-randomized controlled studies included in this meta-analysis was assessed using the NOS. The quality scores of all included studies in the analysis were ≥6 (9 points for 8 studies, 8 points for 14 studies, 7 points for 19 studies, and 6 points for the remaining 3 studies), meeting the requirements for conducting a meta-analysis. Detailed scoring criteria can be found in Supplementary Table 1.

### Incidence rate

The incidence of UTI in patients with hip fractures was reported in 42 included studies [2 articles (12, 13) only reported risk factors and did not provide incidence data]. The pooled incidence of UTI in hip fracture patients was 11% (95% CI: 0.08–0.14) (Supplementary Figure 1). We observed significant heterogeneity among the studies (I^2^ = 99.97%, *P* < 0.01). To explore the source of heterogeneity, a meta-regression was conducted. The meta-regression results indicated that the percentage of female patients (*P* < 0.001), year of publication (*P* < 0.02), and region (*P* = 0.018) were significantly associated with heterogeneity. However, sample size (*P* = 0.213), and study type (*P* = 0.593) were not identified as the sources of heterogeneity. The details can be found in Supplementary Table 2.

### Potential risk factors

**Figure 2:**
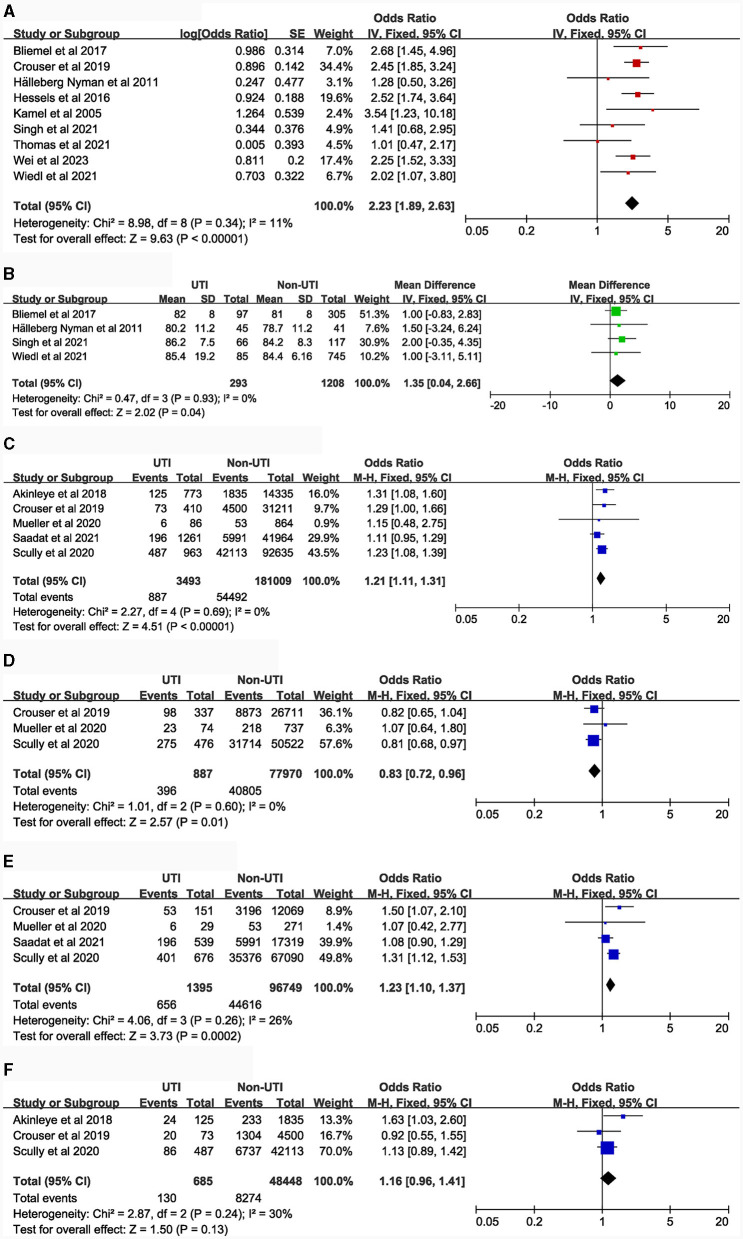
Forest plot for Demographics**. (A)**, Gender; **(B)**, Age (continuous data); **(C)**, BMI (≥30.0 kg/m^2^ vs. <30.0 kg/m^2^); **(D)**, BMI (Overweight VS. Normal weight); **(E)**, Body Mass Index (Obesity VS. Overweight); **(F)**, BMI (Morbid obesity vs. Obesity). CI, Confidence Interval; df, Degrees of Freedom; M-H, Mantel-Haenszel.

**Figure 3:**
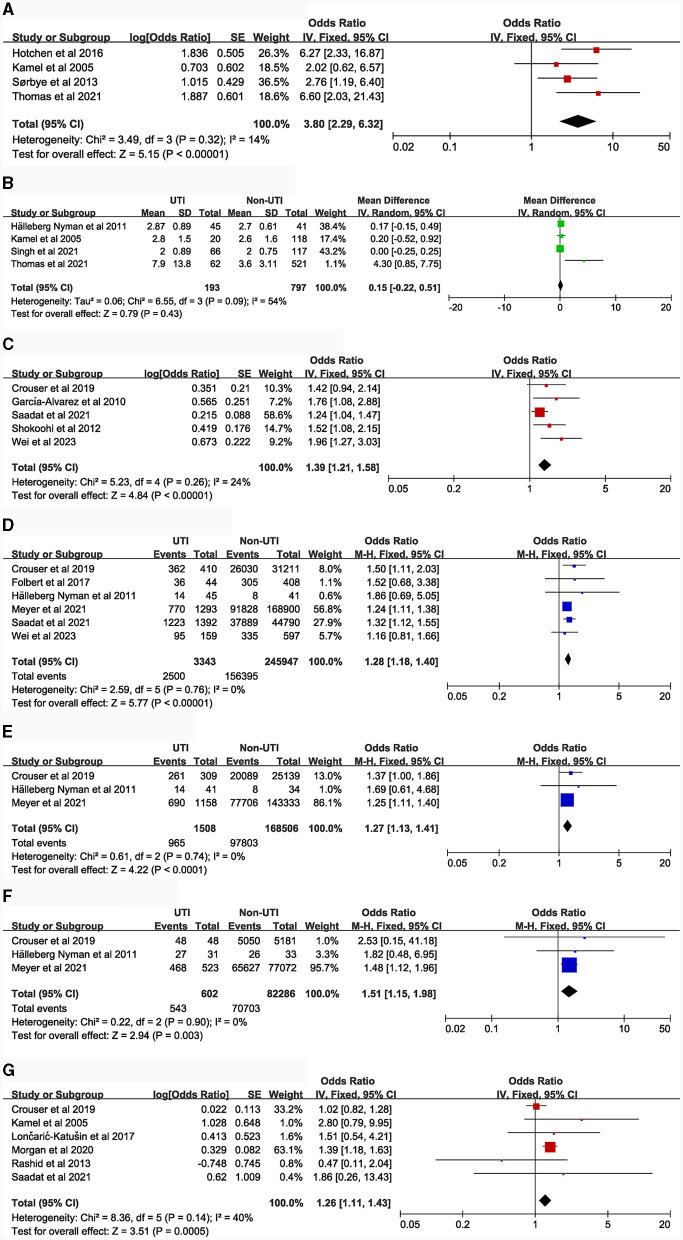
Forest plots for Admission treatment and Anesthesia-related factors. **(A)**, Catheterization; **(B)**, Total time with urinary catheter; **(C)**, Blood transfusion; **(D)**, American Society of Anesthesiologists (≥III vs. <III); **(E)**, ASA (III vs. II); **(F)**, ASA (II vs. I); **(G)**, Type of anesthesia (General anesthesia vs. Spinal anesthesia).

Table 1 details the main characteristics of the 44 items included in the study, while Table 2 presents the specific results of the meta-analysis. The following risk factors are crucial for clinical doctors to identify and intervene in the occurrence of UTI: female [9 studies (5, 14–21), OR = 2.23, 95% CI: 1.89–2.63, Figure 2A]; urinary catheterization [4 studies (18, 19, 22, 23), OR = 3.8, 95% CI: 2.29–6.32, Figure 3A]; delirium [6 studies (12, 13, 18, 24–26), OR = 2.66, 95% CI: 2.05–3.47, Figure 5A]; dementia [5 articles (5, 14, 18, 27, 28), OR = 1.82, 95% CI: 1.62–2.06, Figure 5B]; history of sepsis [2 articles (7, 15), OR = 7.13, 95% CI: 5.51–9.22, Figure 6A].

**Table 1:** Characteristics of included studies.

**References**	**Year**	**Country**	**Study type**	**Sample size**		**Risk factors**	NOS^*^score^**^
				**Total**	**UTI**		
Hälleberg et al. (16)	2011	Sweden	Cohort	86	45	Gender, Age, Catheterization, ASA^*^, Type of fracture, Time to Surgery, Neoplasm, Albumin	9
Hessels et al. (17)	2016	USA^*^	Case-control	2,021	181	Gender, Time to surgery	9
Saadat et al. (7)	2021	USA	Case-control	46,263	1,397	Gender, BMI^*^, Blood transfusion, ASA, Type of anesthesia, Operative time, Hypertension, CHF^*^, Neoplasm, Steroid, COPD^*^, Sepsis, Albumin	9
Wiedl et al. (21)	2021	Germany	Cohort	830	85	Gender, Age	8
Singh et al. (5)	2021	USA	Case-control	183	66	Gender, Age, Catheterization, LOS^*^, Dementia, Hypertension, CHF, Neoplasm, Steroid, COPD	9
Kamel et al. (18)	2005	USA	Cohort	138	20	Gender, Catheterization, Type of anesthesia, Time to Surgery, Time to Surgery, LOS, Diabetes, Delirium, Dementia, Albumin	9
Wei et al. (20)	2023	China	Case-control	756	159	Gender, Blood transfusion, ASA, Type of fracture, Diabetes, Hypertension, Neoplasm	9
Thomas et al. (19)	2021	Canada	Case-control	583	62	Gender, Catheterization	8
Crouser et al. (15)	2019	USA	Cohort	31,621	410	Gender, BMI, Blood transfusion, ASA, Type of anesthesia, Type of surgery, Operative time, Diabetes, Hypertension, CHF, Neoplasm, Steroid, COPD, Sepsis	9
Bliemel et al. (14)	2017	Germany	Cohort	402	97	Gender, Age, Type of fracture, LOS, Diabetes, Dementia, Parkinson's disease	9
Müller et al. (30)	2020	Germany	Cohort	950	86	BMI	6
Scully et al. (31)	2020	USA	Cohort	93,598	963	BMI	8
Akinleye et al. (29)	2018	USA	Cohort	15,108	773	BMI	7
Hotchen et al. (22)	2016	UK^*^	Cohort	207	36	Catheterization	7
Sørbye et al. (23)	2013	Norway	Case-control	331	42	Catheterization	8
Shokoohi et al. (32)	2012	UK	Case-control	919	168	Blood transfusion	7
Folbert et al. (34)	2017	Netherlands	Case-control	452	44	ASA	8
Meyer et al. (33)	2021	Sweden	Cohort	170,193	1,293	ASA	7
Morgan et al. (36)	2020	UK	Case-control	8,144	812	Type of anesthesia	8
Lončarić et al. (35)	2017	Croatia	Case-control	115	23	Type of anesthesia	7
Rashid et al. (37)	2013	Pakistan	Cohort	194	8	Type of anesthesia	7
Ng et al. (38)	2023	Singapore	Cohort	1,524	154	Type of fracture	6
de Lima et al. (39)	2021	Brazil	Cohort	376	42	Type of fracture	7
Dawson et al. (41)	2018	UK	Cohort	92	3	Type of surgery	8
Huang et al. (42)	2023	China	Case-control	547,250	15,114	Type of surgery	7
Anthony et al. (40)	2017	USA	Cohort	4,215	231	Type of surgery, Time to Surgery	7
Liodakis et al. (43)	2016	Canada	Cohort	4,058	256	Type of surgery	8
Miller et al. (44)	2014	USA	Case-control	1,202	77	Type of surgery	7
Vidán et al. (53)	2011	Spain	Cohort	2,250	231	Time to Surgery	7
Glassou et al. (52)	2019	Danish	Case-control	72,520	4,205	Time to Surgery	7
Tian et al. (51)	2020	China	Cohort	644	18	Diabetes	8
Martinez et al. (50)	2017	Spanish	Case-control	115,234	279	Diabetes	8
Ekström et al. (48)	2013	Sweden	Cohort	2,133	493	Diabetes	8
Golinvaux et al. (49)	2015	USA	Case-control	9,938	614	Diabetes	7
Panteli et al. (26)	2021	UK	Case-control	519	75	Delirium	7
Plaza et al. (12)	2020	Spain	Cohort	287	NA^*^	Delirium	8
Rajeev et al. (13)	2022	UK	Case-control	598	NA	Delirium	6
Morandi et al. (24)	2019	Italy	Cohort	519	136	Delirium	7
Muangpaisan et al. (25)	2015	Thailand	Case-control	80	18	Delirium	8
García et al. (27)	2010	Spain	Cohort	290	94	Blood transfusion, Dementia	7
Tsuda et al. (28)	2015	Japan	Case-control	87,654	2,163	Dementia	7
Nguyen et al. (47)	2022	Denmark	Cohort	77,550	2,741	Parkinson's disease	8
Huang et al. (45)	2015	China	Cohort	397,766	84,472	Parkinson's disease	7
Mathew et al. (46)	2013	Czech Republic	Cohort	25	10	Parkinson's disease	7

^*^USA, United States of America; UK, United Kingdom; ASA, American Society of Anesthesiologists; BMI, Body Mass Index; COPD, Chronic Obstructive Pulmonary Disease; CHF, Congestive Heart Failure; LOS, Length of hospital stays; NA, Not Available; NOS, Newcastle-Ottawa Quality Assessment Scale. ^**^The detailed assessment of study quality can be found in the Appendix.

**Table 2:** Results of the meta-analysis.

**Factors**	**Data type**	**I^2^ (%)**	**Q-test (P)**	OR^*^	95% CI^*^	***P*-value**	**Analysis model**
**Demographics**							
Female	Dichotomous	11	0.34	2.23	1.89–2.63	<0.01	Fixed
Age, years	Continuous	0	0.93	1.35	0.04–2.66	0.04	Fixed
**BMI** ^*^							
≥30.0 kg/m^2^ vs. < 30.0kg/m^2^	Dichotomous	0	0.69	1.21	1.11–1.31	<0.01	Fixed
Overweight vs. normal weight	Dichotomous	0	0.60	0.83	0.72–0.96	0.01	Fixed
Obesity vs. overweight	Dichotomous	26	0.26	1.23	1.10–1.37	<0.01	Fixed
Morbid obesity vs. obesity	Dichotomous	30	0.24	1.16	0.96–1.41	0.13	Fixed
**Admission treatment-related factors**							
Catheterization	Dichotomous	14	0.32	3.80	2.29–6.32	<0.01	Fixed
Total time with urinary catheter	Continuous	54	0.09	0.15	−0.22–0.51	0.43	Random
Blood transfusion	Dichotomous	24	0.26	1.39	1.21–1.58	<0.01	Fixed
**Anesthesia-related factors**							
**ASA** ^*^							
ASA ≥ III VS. ASA < III	Dichotomous	0	0.76	1.28	1.18–1.40	<0.01	Fixed
ASA III VS. ASA II	Dichotomous	0	0.74	1.27	1.13–1.41	<0.01	Fixed
ASA II VS. ASA I	Dichotomous	0	0.90	1.51	1,15–1.98	<0.01	Fixed
Type of anesthesia	Dichotomous	40	0.14	1.26	1.11–1.43	<0.01	Fixed
**Surgical-related factors**							
Type of fracture	Dichotomous	28	0.24	1.25	1.01–1.54	0.04	Fixed
Type of surgery	Dichotomous	27	0.23	0.70	0.59–0.84	<0.01	Fixed
Operative time	Dichotomous	0	0.38	0.92	0.83–1.01	0.07	Fixed
**Time to Surgery**							
Time to Surgery, hours	Continuous	12	0.29	1.20	−1.58–3.98	0.40	Fixed
Time > 48 h vs. Time ≤ 48 h	Dichotomous	73	0.005	1.04	0.89–1.21	0.64	Random
Length of hospital stays, days	Continuous	18	0.30	1.44	0.66–2.23	<0.01	Fixed
**Comorbidities**							
Delirium	Dichotomous	36	0.17	2.66	2.05–3.47	<0.01	Random
Dementia	Dichotomous	0	0.46	1.82	1.62–2.06	<0.01	Fixed
Parkinson's disease	Dichotomous	24	0.27	1.53	1.46–1.61	<0.01	Fixed
Diabetes	Dichotomous	42	0.10	1.27	1.13–1.43	<0.01	Fixed
Hypertension	Dichotomous	35	0.20	1.14	1.03–1.26	<0.01	Fixed
CHF^*^	Dichotomous	0	0.70	1.35	1.10–1.66	<0.01	Fixed
History of sepsis	Dichotomous	13	0.28	7.13	5.51–9.22	<0.01	Fixed
Neoplasm	Dichotomous	11	0.34	0.82	0.60–1.13	0.23	Fixed
COPD^*^	Dichotomous	4	0.35	1.12	0.97–1.29	0.11	Fixed
Chronic steroid use	Dichotomous	0	0.89	1.29	1.06–1.57	0.01	Fixed
**Laboratory tests**							
Albumin, g/l	Continuous	0	1.00	0.00	−0.26–0.26	1.00	Fixed

^*^OR, odds ratio; CI, confidence interval; ASA, American Society of Anesthesiologists; BMI, Body Mass Index; COPD, Chronic Obstructive Pulmonary Disease; CHF, Congestive Heart Failure.

**Figure 4:**
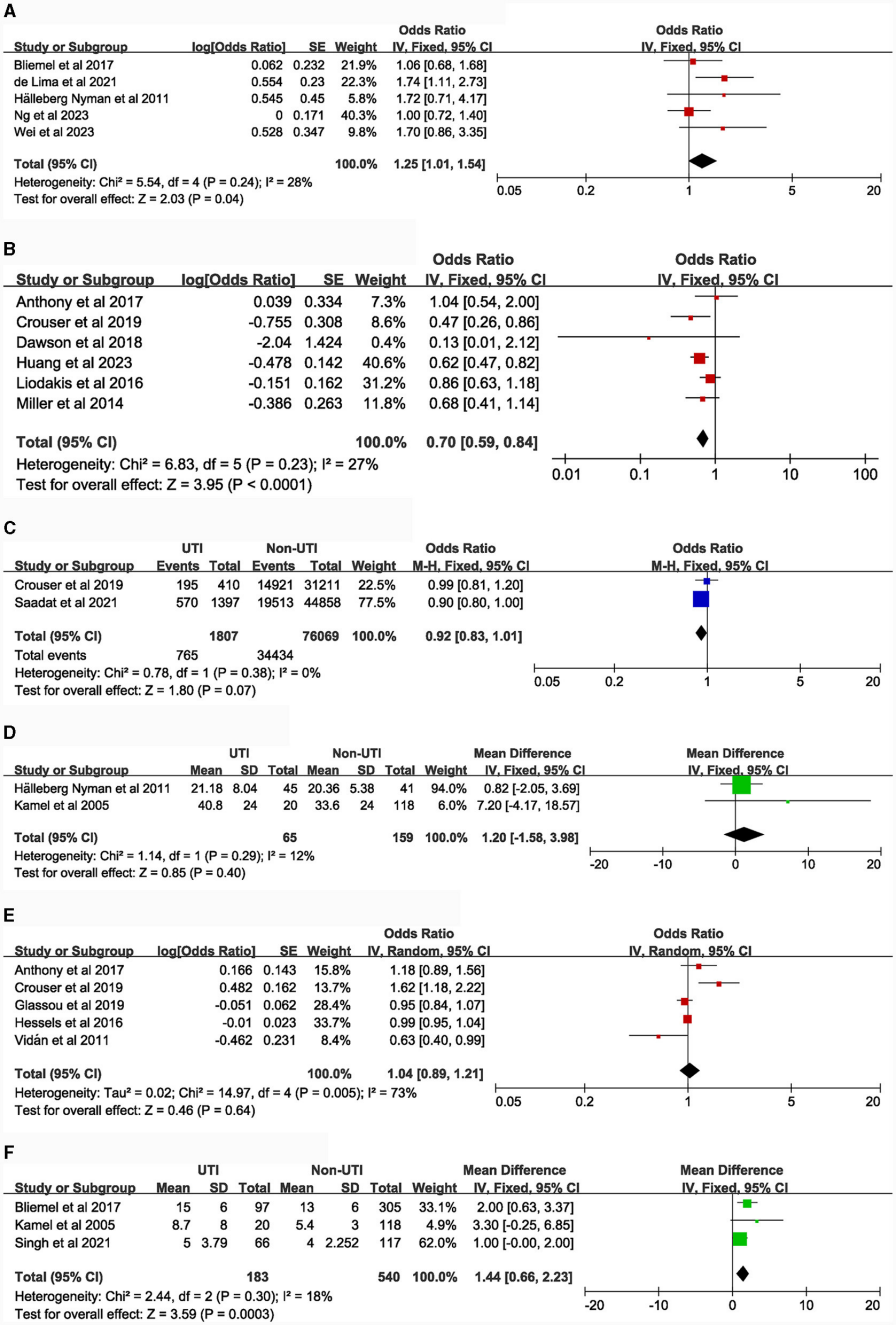
Forest plots for surgical-related factors. **(A)**, Type of fracture (intertrochanteric fracture vs. femoral neck fracture); **(B)**, type of surgery (total hip replacement vs. hemiarthroplasty); **(C)**, Operative time (>1 h vs. ≤ 1 h); **(D)**, Time to Surgery (continuous data); **(E)**, Time to Surgery (>48 h vs. ≤ 48 h); **(F)**, Length of hospital stays (continuous data).

**Figure 5:**
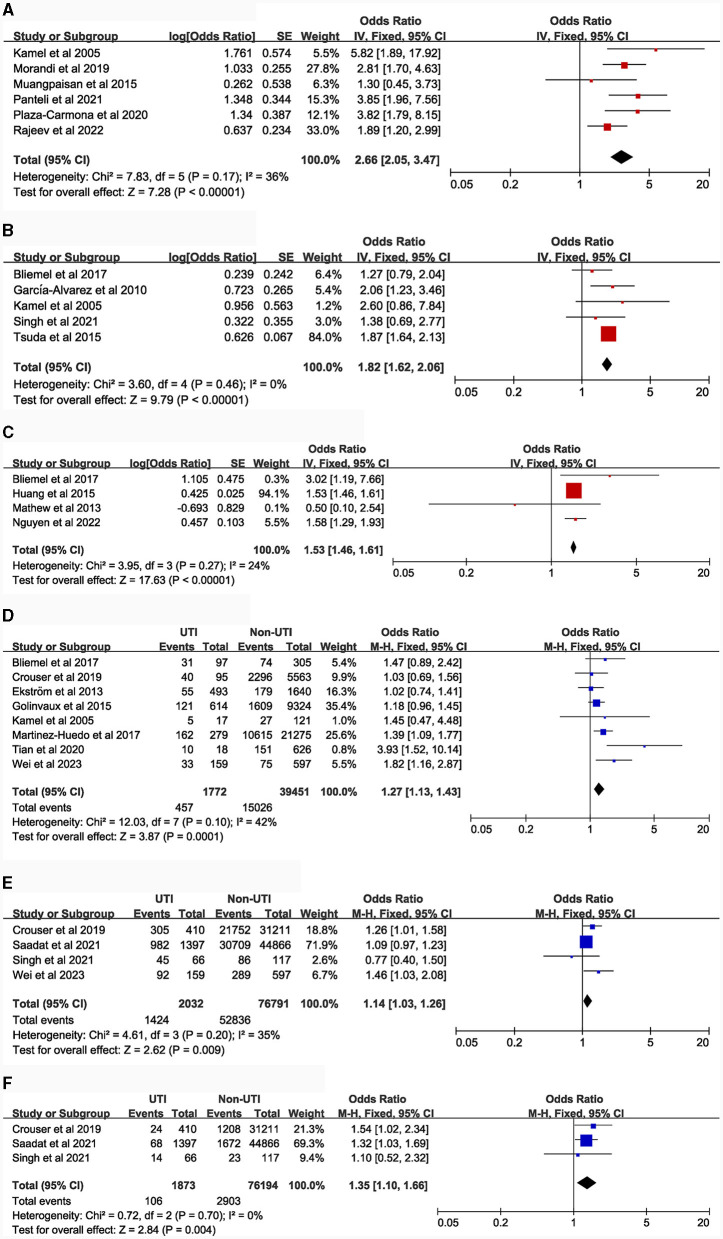
Forest plots for comorbidities. **(A)**, delirium; **(B)**, dementia; **(C)**, Parkinson's disease; **(D)**, diabetes; **(E)**, hypertension; **(F)**, congestive heart failure.

**Figure 6:**
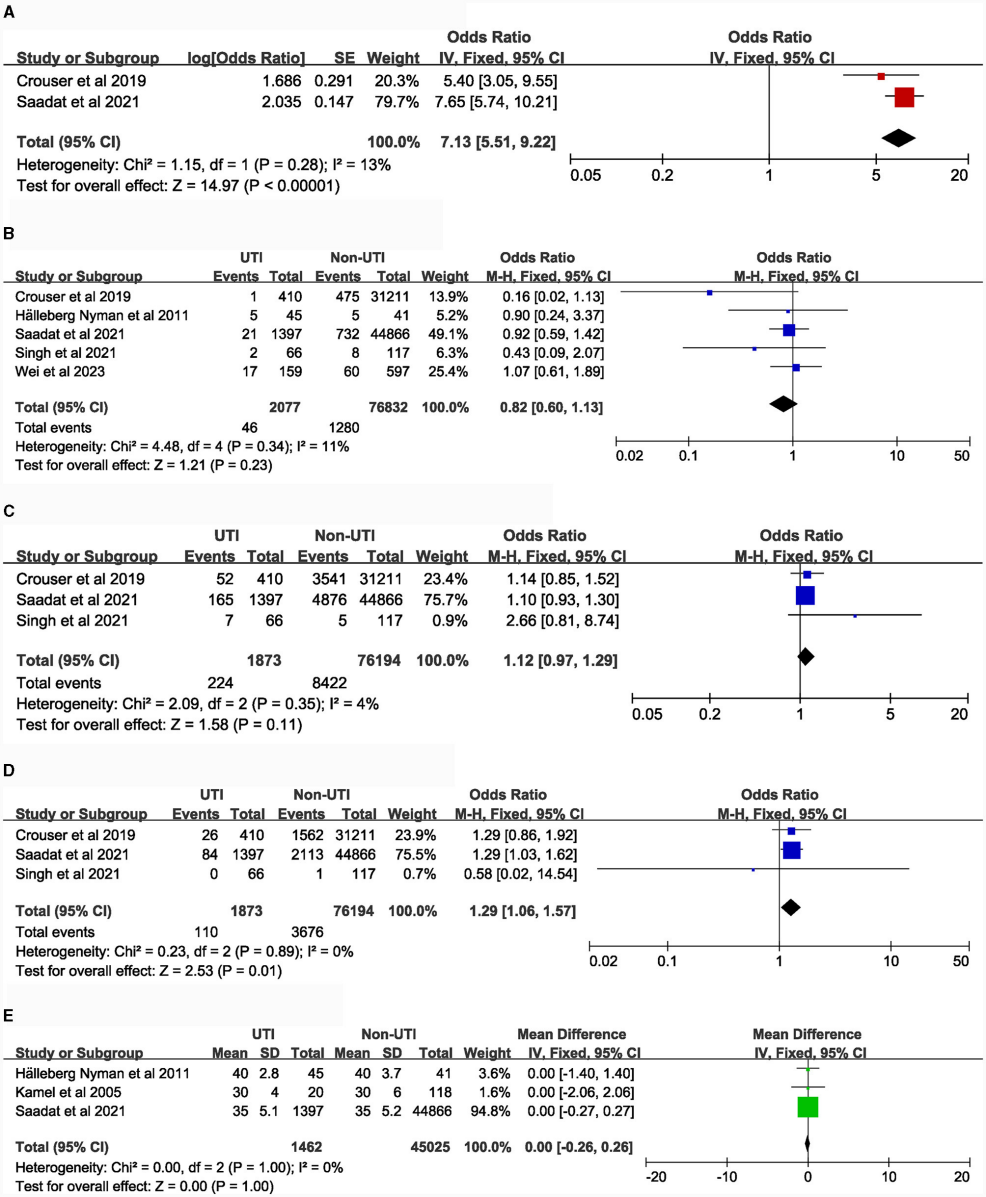
Forest plots for comorbidities and laboratory tests. **(A)**, history of sepsis; **(B)**, neoplasm; **(C)**, chronic obstructive pulmonary disease; **(D)**, chronic steroid use; **(E)**, preoperative albumin.

The following risk factors hold moderate clinical significance and warrant attention in clinical practice: advanced age [4 studies (5, 14, 16, 21), MD = 1.35, 95% CI: 0.04–2.66, Figure 2B]; Body Mass Index ≥ 30.0 kg/m^2^ [5 studies (7, 15, 29–31), OR = 1.21, 95% CI: 1.11–1.31, Figure 2C]; blood transfusion [5 studies (7, 15, 20, 27, 32), OR = 1.39, 95% CI: 1.21–1.58, Figure 3C]; American Society of Anesthesiologists classification ≥ III [6 articles (7, 15, 16, 20, 33, 34), OR = 1.28, 95% CI: 1.18–1.40, Figure 3D]; general anesthesia [6 studies (7, 15, 18, 35–37), OR = 1.26, 95% CI: 1.11–1.43, Figure 3G]; intertrochanteric fractures [compared to femoral neck fractures, 5 articles (14, 16, 20, 38, 39), OR = 1.25, 95% CI: 1.01–1.54, Figure 4A]; hemiarthroplasty [compared to total hip replacement, 6 studies (15, 40–44), OR = 1.43, 95% CI: 1.19–1.69, Figure 4B]; length of hospital stay [3 articles (5, 14, 18), MD = 1.44, 95% CI: 0.66–2.23, Figure 4F]; Parkinson's disease [4 articles (14, 45–47), OR = 1.53, 95% CI: 1.46–1.61, Figure 5C]; diabetes [8 articles (14, 15, 18, 20, 48–51), OR = 1.27, 95% CI: 1.13–1.43, Figure 5D]; hypertension [4 articles (5, 7, 15, 20), OR = 1.14, 95% CI: 1.03–1.26, Figure 5E]; congestive heart failure [3 articles (5, 7, 15), OR = 1.35, 95% CI: 1.10–1.66, Figure 5F]; and long-term steroid use [3 studies (5, 7, 15), OR = 1.29, 95% CI: 1.06–1.57, Figure 6D]. Additionally, we conducted a more detailed analysis by categorizing high BMI (Body Mass Index) into different groups according to guidelines: Normal weight (BMI: 18.5–24.9 kg/m^2^), Overweight (BMI: 25–29.9 kg/m^2^), Obesity (BMI: 30–39.9 kg/m^2^), and Morbid Obesity (BMI ≥ 40 kg/m^2^). The comparison between groups revealed that obese patients with hip fractures had an increased risk of UTI compared to overweight patients (OR = 1.23, 95% CI: 1.10–1.37, Figure 2E). No significant difference was observed between morbid obesity and obesity groups (*P* > 0.05, Figure 2F). Surprisingly, overweight patients had lower UTI risk than normal-weight patients (OR = 0.83, 95% CI: 0.72–0.96, Figure 2D). Similarly, our findings indicate a direct association between the incremental rise in ASA (American Society of Anesthesiologists) classification and the heightened risk of UTI occurrence (*P* < 0.05, Figures 3E, F).

There was no observed correlation between the following risk factors and UTI in patients with hip fractures: duration of catheterization [4 studies (5, 16, 18, 19), Figure 3B]; surgical duration longer than 1 h [2 articles (7, 15), Figure 4C]; time to surgery [dichotomous and continuous variables, 7 articles (15–18, 40, 52, 53), Figures 4D, E]; neoplasm [5 articles (5, 7, 15, 16, 20), Figure 6B]; chronic obstructive pulmonary disease [3 studies (5, 7, 15), Figure 6C]; preoperative albumin levels [3 articles (7, 16, 18), Figure 6E].

### Sensitivity analyses

It was observed that the effect size of the gender risk factor lacked robustness (Supplementary Figure 3a). Consequently, the study (7) conducted by Saadat et al. was excluded from the investigation concerning the relationship between gender and UTI in patients with hip fractures. However, the effect sizes of the remaining risk factors demonstrated consistent stability during sensitivity tests. Supplementary Figures 3, 4 provide the detailed results of these sensitivity tests.

### Publication bias

A funnel plot was examined for visual inspection, and quantitative assessments using Begg's test and Egger's test were conducted. The results indicated no evidence of publication bias among the included studies (Supplementary Figures 5, 6).

## Discussion

As the sole systematic review investigating the risk factors for UTIs in hip fracture patients, this study synthesizes findings from forty-four articles concerning UTI incidence and associated risk factors in this patient population. The primary results are as follows: UTI emerges as a prevalent complication following hip fracture, with an incidence rate reaching up to 11% (95% CI: 8%−14%), consistent with findings reported across multiple studies (54, 55), and influenced by the proportion of female patients in the sample, year of publication, and regional variations. We explore a total of twenty-four potential risk factors, eighteen of which demonstrate significance. While some risk factors may warrant further investigation, our review represents the most current and inclusive assessment available at present.

The majority of studies (14, 15, 17, 20, 21) have identified females as a potential independent risk factor for UTI in hip fracture patients, a finding consistent with the final conclusion of this study. The susceptibility of females to UTI is primarily attributed to the anatomical structure of their short urethra, which is located close to the anus, thereby increasing the risk of bacterial colonization. This unique physiological structure predisposes women to UTI, which is further exacerbated by the decline in estrogen levels after menopause. Recent studies have demonstrated that specific estrogen receptors are expressed on urethral epithelial cells, and the reduction of postmenopausal estrogen alters the immune status of the urethral mucosa, leading to an increased risk of UTI. Hormone replacement therapy is therefore recommended for the prevention and treatment of UTIs in postmenopausal women (56).

This systematic review suggests that elderly hip fracture patients should be cautious about the occurrence of UTI. With the decline of physical function, immune system, bladder urine retention (due to prostate hyperplasia and relaxation of the bladder detrusor muscle), the risk of UTI increases significantly in the elderly population (57). Furthermore, hip fracture patients who have lost their ability to stand and perform self-care may experience a decrease in urinary flow rate and an increase in residual urine, thereby increasing the risk of UTI (58).

Most studies (15, 29, 31) have reported a potential correlation between high body mass index (BMI ≥ 30.0 kg/m^2^) and UTI in hip fracture patients, suggesting that obese individuals are more susceptible to UTIs. However, the specific mechanisms underlying this association remain unclear. Some articles have proposed that the link between obesity and UTI susceptibility may be attributed to reduced immune response (59, 60). In subgroup analysis, we observed that overweight hip fracture patients did not exhibit a higher risk of UTI compared to patients with normal weight. This finding aligns with the conclusion of Alhabeeb's systematic review (59) on the relationship between BMI and UTI. However, obese hip fracture patients were not as fortunate, as their risk of UTI was 1.23 times higher than that of overweight patients. Moreover, the risk of UTI did not further increase in the morbidly obese hip fracture population.

The question of whether catheterized patients are more prone to UTIs has long been of interest. Kamel's study (18) indicated that the use of catheters may not be associated with an increased risk of UTI, as catheters can alleviate urinary retention and reduce residual urine volume. Conversely, Thomas's study (19) suggested that catheterization could elevate the risk of UTI in hip fracture patients and recommended prompt removal or reevaluation of catheter benefits within 24 h after surgery. Our study provides stronger evidence supporting the occurrence of UTIs in hip fracture patients due to catheterization. This can be explained by the mucosal damage caused by catheter insertion, which provides an opportunity for bacterial colonization due to insufficient urine flushing. Our study emphasizes the importance of thoroughly assessing the individual benefits and risks of catheterization for patients, rather than routine preoperative or postoperative catheterization. Additionally, the lack of differentiation between indwelling and intermittent catheterization in the included studies, as well as the limited number of studies, may have contributed to the absence of significant differences in conclusions regarding the total duration of catheterization. Several studies (7, 20, 27, 32) unanimously suggest a potential relationship between receiving allogeneic red blood cell transfusion during hospitalization and UTIs in hip fracture patients. However, the underlying mechanism linking transfusion and UTI remains unclear, although current research indicates that the host's immune defense undergoes changes after transfusion.

The ASA identifies high-risk patients by assessing comorbidities and other health issues (34). Saadat (7, 34) observed that patients with hip fractures and an ASA score ≥III are more likely to develop UTIs than those with an ASA score of I-II. This finding is consistent with our overall conclusion, suggesting that patients with higher ASA scores may be more susceptible to UTIs. A higher ASA score signifies poorer physical function, debilitation leading to longer bedridden periods, and reduced self-care ability, which may contribute to the increased risk of UTIs.

The impact of anesthesia type on postoperative UTI in hip fracture patients remains controversial. While Morgan's study (36) suggested a possible correlation between general anesthesia and postoperative UTI, Rashid (37) did not support this conclusion. Some scholars even argue that spinal anesthesia may be more likely to lead to postoperative UTI. Our summary results indicate that hip fracture patients who undergo general anesthesia may have a higher risk of developing postoperative UTIs.

Surgical risk factors highlight the need to identify and intervene in high-risk patients for UTI before and during surgery. de Lima (39) reported an increased risk of UTI in patients with intertrochanteric fractures compared to those with femoral neck fractures, although Bliemel's research (14) did not find a corresponding difference. Regarding surgical type, patients undergoing hemiarthroplasty (HHA) appeared significantly older, resulting in a higher incidence of UTI in such patients. This challenges the fixed mindset that total hip replacement surgery carries a higher risk of complications due to its larger trauma and longer postoperative recovery time. The summarized results suggest that we should pay more attention to elderly patients receiving hemiarthroplasty. UTI risk was not significantly affected at the 1-h surgical time point, and it was not found that early surgery recommended in guidelines could reduce complications, possibly due to limited inclusion studies. Finally, our results suggest that hip fracture patients with longer hospital stays may have a higher risk of developing UTIs, which is consistent with the concept of early discharge and recovery.

With advancing research, an expanding body of literature has established a strong correlation between pre-existing comorbidities and postoperative UTIs in hip fracture patients. Recent studies have specifically focused on the association between delirium, dementia, Parkinson's disease, and UTIs. In hip fracture patients, these conditions are characterized by cognitive impairment or acute confusional states, leading to a loss of self-care ability that significantly increases the risk of postoperative UTIs. The influence of diabetes on UTIs after hip fracture is explained by the role of hyperglycemia in compromising the immune system and facilitating bacterial invasion, although the exact underlying mechanism requires further exploration (20). Patients with a medical history of sepsis or prolonged steroid use who are currently suffering from hip fractures may be at an increased risk of developing postoperative UTIs due to immunodeficiency. However, conflicting evidence exists regarding the impact of hypertension and congestive heart failure on UTI susceptibility in hip fracture patients. Therefore, the association between cardiovascular disease and UTI warrants further investigation to elucidate potential mechanisms. Additionally, no significant correlation was found between UTIs and hip fracture patients with COPD or tumors. Serum albumin levels reflect patients' nutritional and immune statuses. However, this study did not establish a conclusive link between preoperative albumin levels and UTI risk.

This study provides recommendations and management strategies to reduce the occurrence of UTIs in patients with hip fractures, based on a systematic review. Firstly, patients admitted due to hip fractures should undergo an assessment of the risk factors for UTIs. For high-risk patients, such as females, older age, obesity, and those with current comorbidities of mental disorders (such as delirium, dementia, Parkinson's) or previous sepsis, it is recommended to enhance urethral care and urine testing during the hospitalization. Additionally, prophylactic use of antibiotics may be considered if necessary. For non-high-risk patients, promoting early mobilization and minimizing hospital stay duration are encouraged in clinical practice to reduce the occurrence of UTIs. Regarding the use of urinary catheters in clinical settings, it is suggested that their removal within a short period or individualized evaluation of their necessity can be beneficial. Special attention should be given to patients undergoing general anesthesia or blood transfusion to prevent UTIs.

## Limitation

This study has several limitations, including the lack of robust evidence from randomized controlled trials and mechanistic studies to definitively identify risk factors. Causal relationships cannot be determined, and all associations should be interpreted as such. Furthermore, the assessment of risk factors relied on limited or contradictory non-randomized controlled trials. Future research should aim to include a more extensive range of studies. Finally, our search was confined to mainstream databases, potentially limiting the breadth of information retrieved.

## Conclusions

This systematic review and meta-analysis demonstrate that UTI is indeed one of the most common complications in hip fracture patients worldwide, with varying incidence rates depending on geographical regions, year of publication and gender distributions. By focusing on the high-risk populations identified in this study, the aim is to achieve clinical prediction, guide early intervention, and implement targeted management.

## Data availability statement

The original contributions presented in the study are included in the article/Supplementary material, further inquiries can be directed to the corresponding authors.

## Author contributions

WW: Conceptualization, Data curation, Formal analysis, Investigation, Methodology, Project administration, Resources, Software, Supervision, Writing – original draft, Writing – review & editing. WY: Investigation, Methodology, Software, Validation, Writing – review & editing. WT: Formal analysis, Investigation, Methodology, Software, Writing – review & editing. YL: Formal analysis, Investigation, Methodology, Writing – review & editing. HS: Conceptualization, Investigation, Methodology, Project administration, Writing – review & editing. WD: Conceptualization, Investigation, Project administration, Software, Writing – review & editing.
