# Supplementary material for: Risk factors for urinary tract infection in geriatric hip fracture patients: a systematic review and meta-analysis
Source: Front Med (Lausanne). 2024 Feb 9;11:1360058. doi: 10.3389/fmed.2024.1360058 (PMC10884186; doi:10.3389/fmed.2024.1360058)
Supplement: Supplementary file 1 [file Data_Sheet_1.docx]

Appendix

**e-Table 1 Assessment of study quality**

| Newcastle-Ottawa Quality Assessment Scale (NOS) | | | | | | | | | |
| --- | --- | --- | --- | --- | --- | --- | --- | --- | --- |
| Study | Selection | | | | Comparability  2 stars | outcomes | | | Total  9 stars |
|  | Representativeness of the exposed cohort  1 star | Selection of the nonexposed cohort  1 star | Ascertainment of exposure  1 star | Demonstration that outcome of interest was not present at start of study  1 star |  | Assessment of outcome  1 star | Follow-up long enough for outcomes to occur  1 star | Adequacy of follow up of cohort  1 star |  |
| Hälleberg et al. [16] | ★ | ★ | ★ | ★ | ★★ | ★ | ★ | ★ | 9 |
| Hessels et al. [17] | ★ | ★ | ★ | ★ | ★★ | ★ | ★ | ★ | 9 |
| Saadat et al. [7] | ★ | ★ | ★ | ★ | ★★ | ★ | ★ | ★ | 9 |
| Wiedl et al. [21] | ★ | ★ | ★ | ★ | ★★ | ★ | ★ |  | 8 |
| Singh et al. [5] | ★ | ★ | ★ | ★ | ★★ | ★ | ★ | ★ | 9 |
| Kamel et al. [18] | ★ | ★ | ★ | ★ | ★★ | ★ | ★ | ★ | 9 |
| Wei et al. [20] | ★ | ★ | ★ | ★ | ★★ | ★ | ★ | ★ | 9 |
| Thomas et al. [19] | ★ | ★ | ★ | ★ | ★★ | ★ |  | ★ | 8 |
| Crouser et al. [15] | ★ | ★ | ★ | ★ | ★★ | ★ | ★ | ★ | 9 |
| Bliemel et al. [14] | ★ | ★ | ★ | ★ | ★★ | ★ | ★ | ★ | 9 |
| Müller et al. [30] | ★ | ★ | ★ |  | ★★ | ★ |  |  | 6 |
| Scully et al. [31] | ★ | ★ | ★ | ★ | ★★ | ★ | ★ |  | 8 |
| Akinleye et al. [29] | ★ | ★ | ★ | ★ | ★★ | ★ |  |  | 7 |
| Hotchen et al. [22] |  | ★ | ★ | ★ | ★★ | ★ | ★ |  | 7 |
| Sørbye et al. [23] | ★ | ★ | ★ | ★ | ★★ | ★ | ★ |  | 8 |
| Shokoohi et al. [32] | ★ | ★ | ★ | ★ | ★★ | ★ |  |  | 7 |
| Folbert et al. [34] | ★ | ★ | ★ | ★ | ★★ | ★ | ★ |  | 8 |
| Meyer et al. [33] | ★ | ★ | ★ | ★ | ★★ | ★ |  |  | 7 |
| Morgan et al. [36] | ★ | ★ | ★ | ★ | ★★ | ★ | ★ |  | 8 |
| Lončarić et al. [35] | ★ | ★ | ★ | ★ | ★★ | ★ |  |  | 7 |
| Rashid et al. [37] | ★ |  | ★ | ★ | ★★ | ★ | ★ |  | 7 |
| Ng et al. [38] | ★ | ★ |  |  | ★ | ★ | ★ |  | 6 |
| de Lima et al. [39] | ★ | ★ | ★ | ★ | ★★ | ★ |  |  | 7 |
| Dawson et al. [41] | ★ | ★ | ★ | ★ | ★★ | ★ | ★ |  | 8 |
| Huang et al. [42] | ★ | ★ |  | ★ | ★★ | ★ | ★ |  | 7 |
| Anthony et al. [40] | ★ | ★ | ★ |  | ★★ | ★ | ★ |  | 7 |
| Liodakis et al. [43] | ★ | ★ | ★ | ★ | ★★ | ★ | ★ |  | 8 |
| Miller et al. [44] | ★ | ★ | ★ | ★ | ★★ | ★ |  |  | 7 |
| Vidán et al. [53] | ★ | ★ | ★ | ★ | ★★ | ★ |  |  | 7 |
| Glassou et al. [52] | ★ | ★ |  | ★ | ★★ | ★ | ★ |  | 7 |
| Tian et al. [51] | ★ | ★ | ★ | ★ | ★★ | ★ | ★ |  | 8 |
| Martinez et al. [50] | ★ | ★ | ★ | ★ | ★★ | ★ |  | ★ | 8 |
| Ekström et al. [48] | ★ | ★ | ★ | ★ | ★ | ★ | ★ |  | 8 |
| Golinvaux et al. [49] | ★ |  | ★ | ★ | ★ | ★ | ★ |  | 7 |
| Panteli et al. [26] | ★ | ★ | ★ | ★ | ★ | ★ |  |  | 7 |
| Plaza et al. [12] | ★ | ★ | ★ | ★ | ★★ | ★ | ★ |  | 8 |
| Rajeev et al. [13] |  | ★ | ★ |  | ★★ | ★ | ★ |  | 6 |
| Morandi et al. [24] | ★ | ★ | ★ | ★ | ★★ | ★ |  |  | 7 |
| Muangpaisan et al. [25] | ★ | ★ | ★ | ★ | ★★ | ★ | ★ |  | 8 |
| García et al. [27] |  | ★ | ★ | ★ | ★★ | ★ | ★ |  | 7 |
| Tsuda et al. [28] | ★ | ★ | ★ | ★ | ★★ | ★ |  |  | 7 |
| Nguyen et al. [47] | ★ | ★ | ★ | ★ | ★ | ★ | ★ |  | 8 |
| Huang et al. [45] | ★ | ★ | ★ | ★ | ★ | ★ |  |  | 7 |
| Mathew et al. [46] | ★ | ★ | ★ | ★ | ★ | ★ |  |  | 7 |

**e-Table 2 Results of Meta-Regression**

| Variable | Coefficient | Standard Error | P-Value |
| --- | --- | --- | --- |
| Gender (%, female) | 0.916 | 0.062 | ＜0.001 |
| Sample size | -0.0001 | 0.0001 | 0.213 |
| Year of publication | -0.008 | 0.004 | 0.020 |
| Study type |  | | |
| Cohort (n=24) |  |  | Reference |
| Case-control (n=18) | -0.015 | 0.029 | 0.593 |
| Region |  | | |
| Europe (n= 20) |  |  | Reference |
| America (n=13) | -0.070 | 0.030 | 0.018 |
| Asia (n=9) | -0.059 | 0.039 | 0.129 |

**
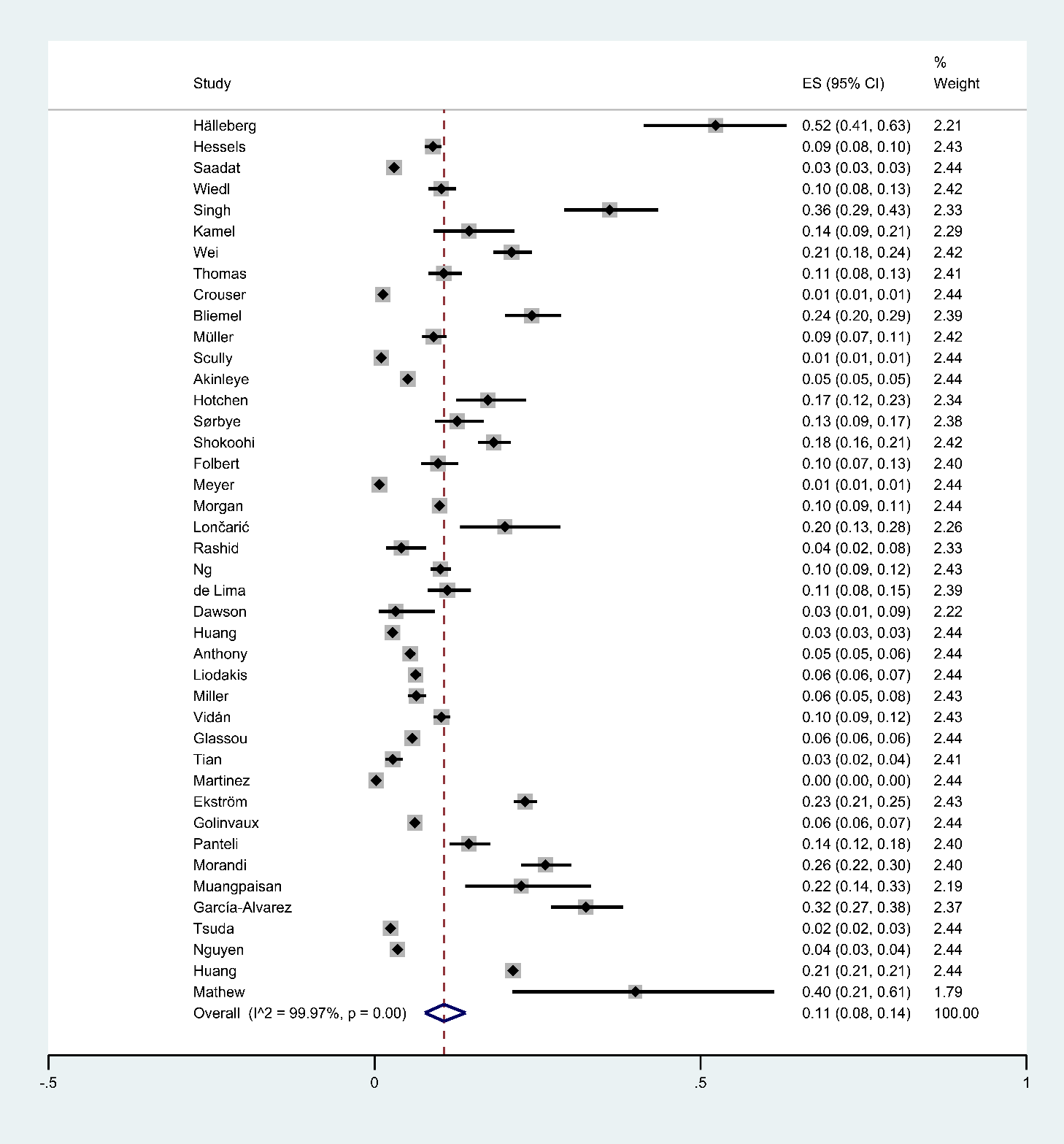
e-Figure 1** The Incidence rate of urinary tract infection in patients with hip fractures (Random effects model)

**
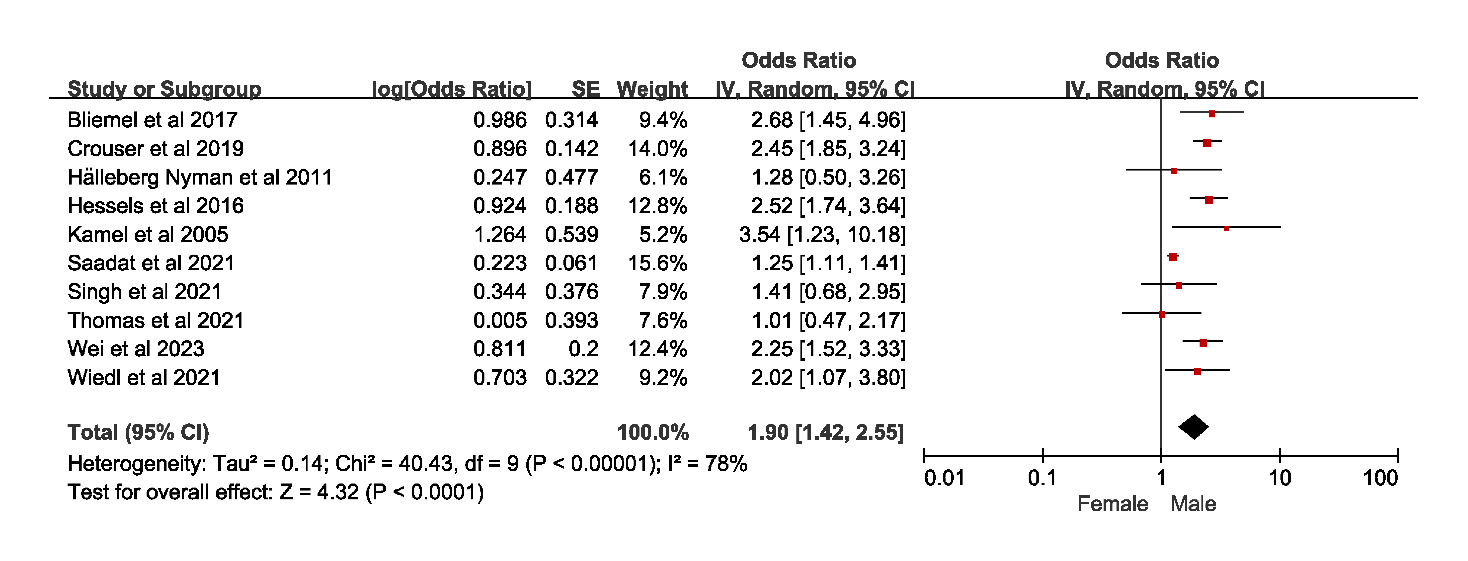
e-Figure 2** Forest plots for gender (Significant Heterogeneity)


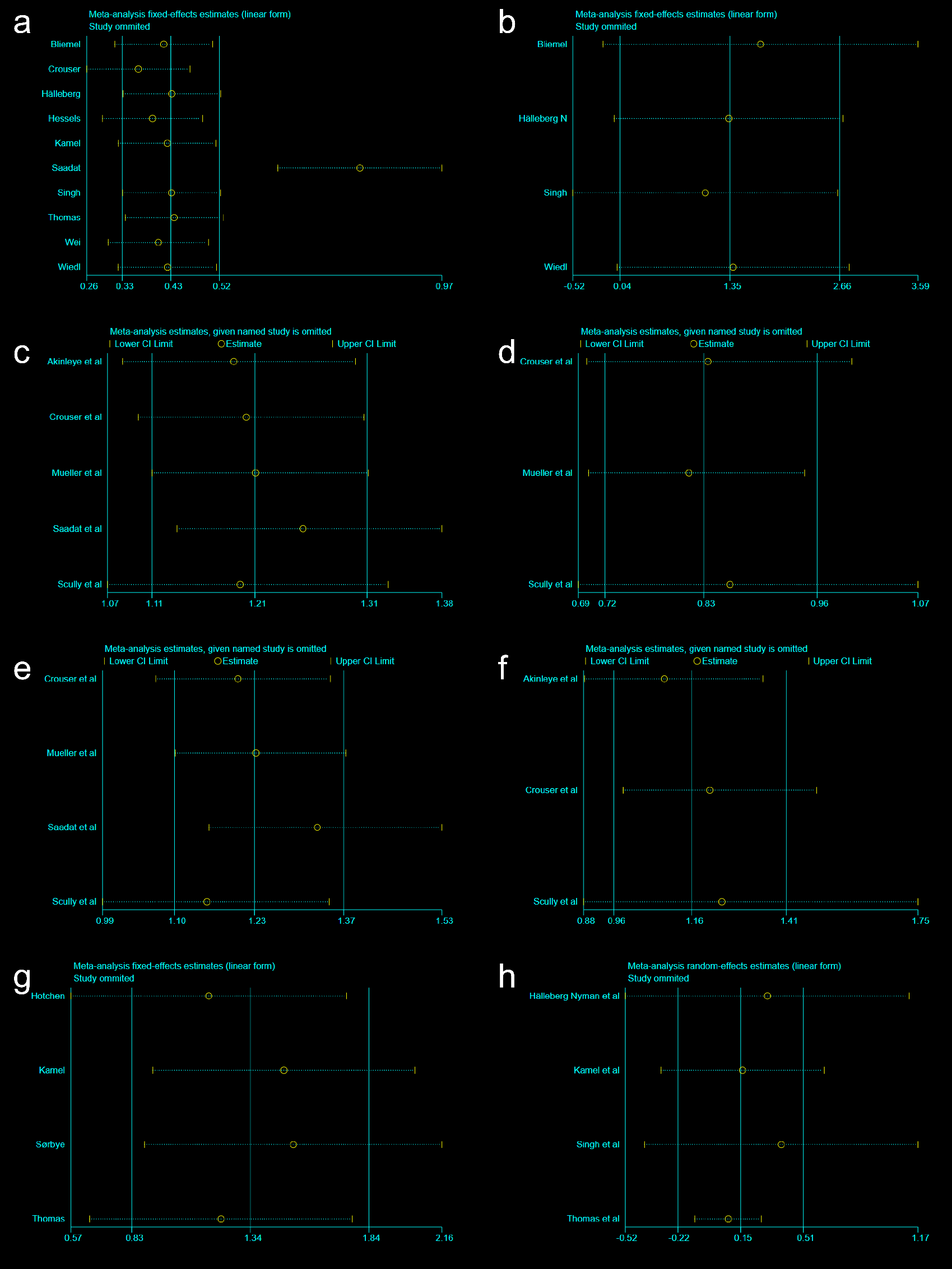


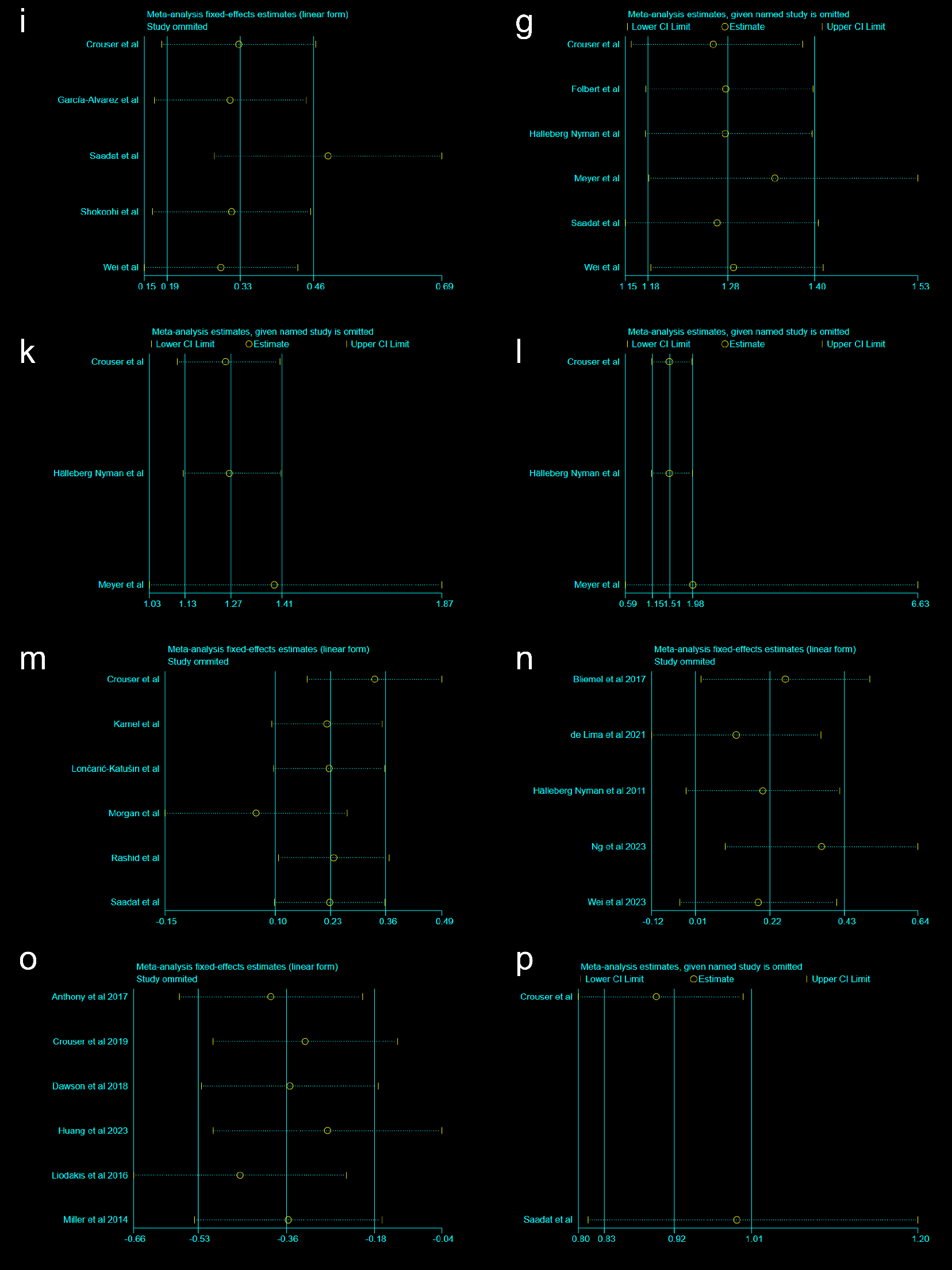


**e-Figure 3** Sensitivity test. **a**, Gender; **b**, Age (Continuous); **c**, BMI (≥30.0 kg/m² VS ＜30.0 kg/m²); **d**, BMI (Overweight VS Normal weight); **e**, BMI (Obesity VS Overweight); **f**, BMI (Morbid obesity VS Obesity); **g**, Catheterization; **h**, Total time with urinary catheter; **i**, Blood transfusion; **j**, ASA (≥III VS＜III); **k**, ASA (III VS II); **l,** ASA (II VS I); **m,** Type of anesthesia; **n,** Type of fracture; **o,** Type of surgery; **p**, Operative time.


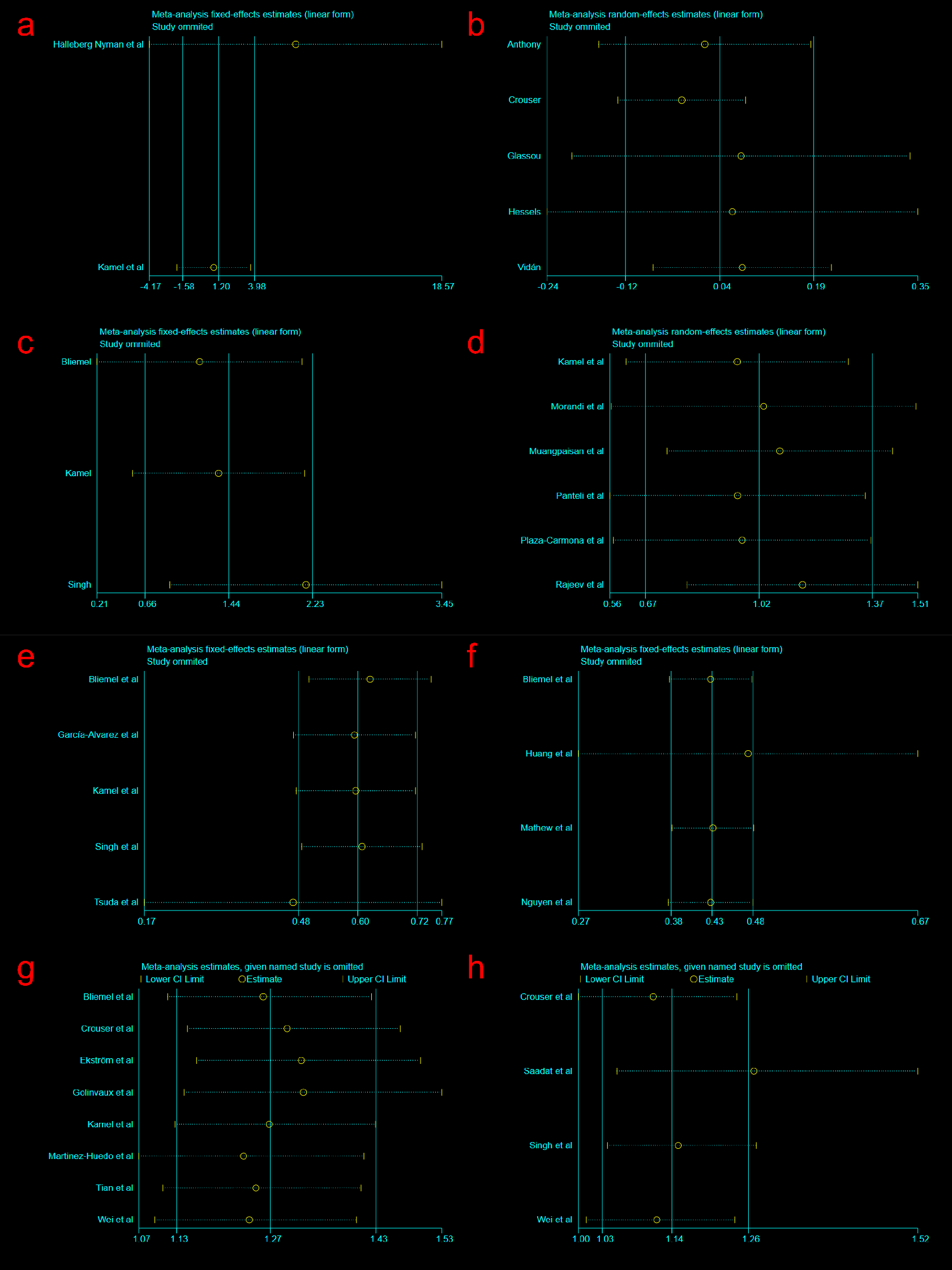


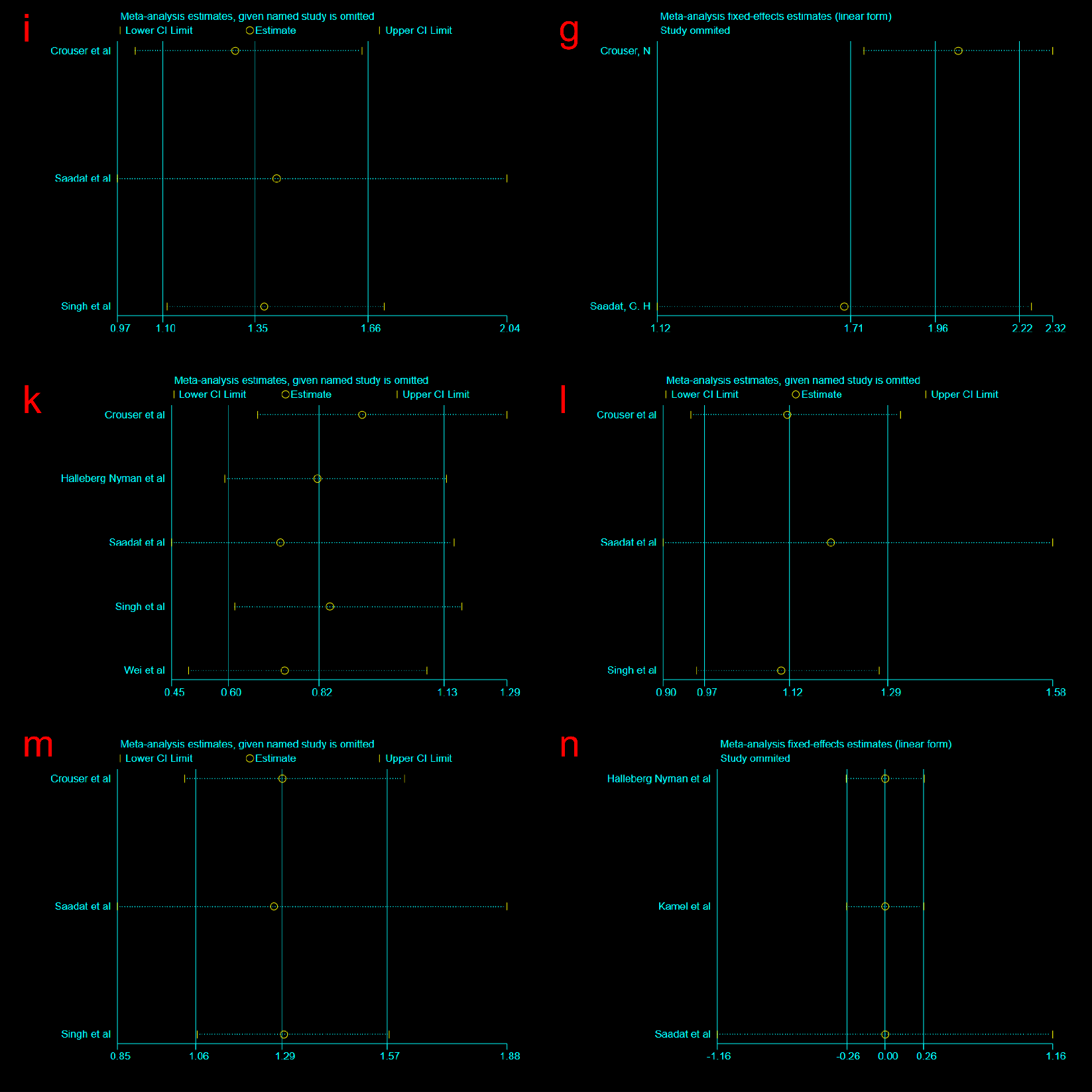


**e-Figure 4** Sensitivity test. **a**, Time to Surgery (Continuous); **b**, Time to Surgery (＞48h VS ≤48h); **c**, Length of hospital stays; **d**, Delirium; **e**, Dementia; **f**, Parkinson’s disease; **g**, Diabetes; **h**, Hypertension; **i**, CHF (Congestive Heart Failure); **j**, History of sepsis; **k**, Neoplasm; **l**, COPD (Chronic Obstructive Pulmonary Disease); **m**, Chronic steroid use; **n**, Albumin.


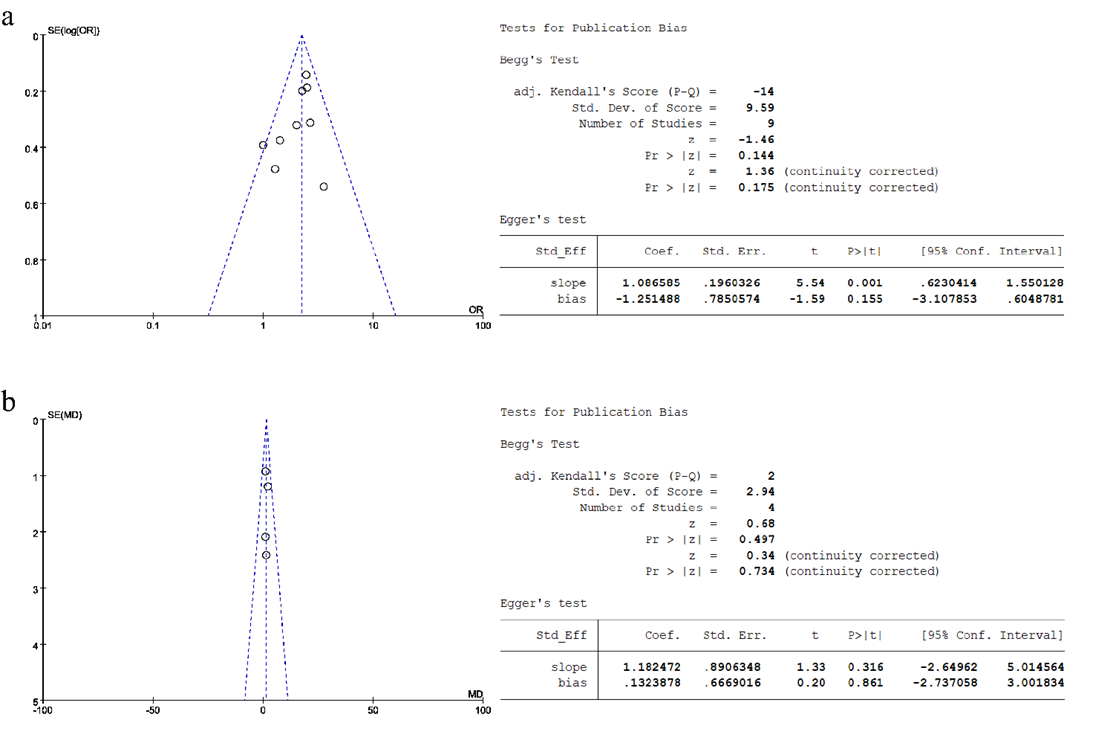


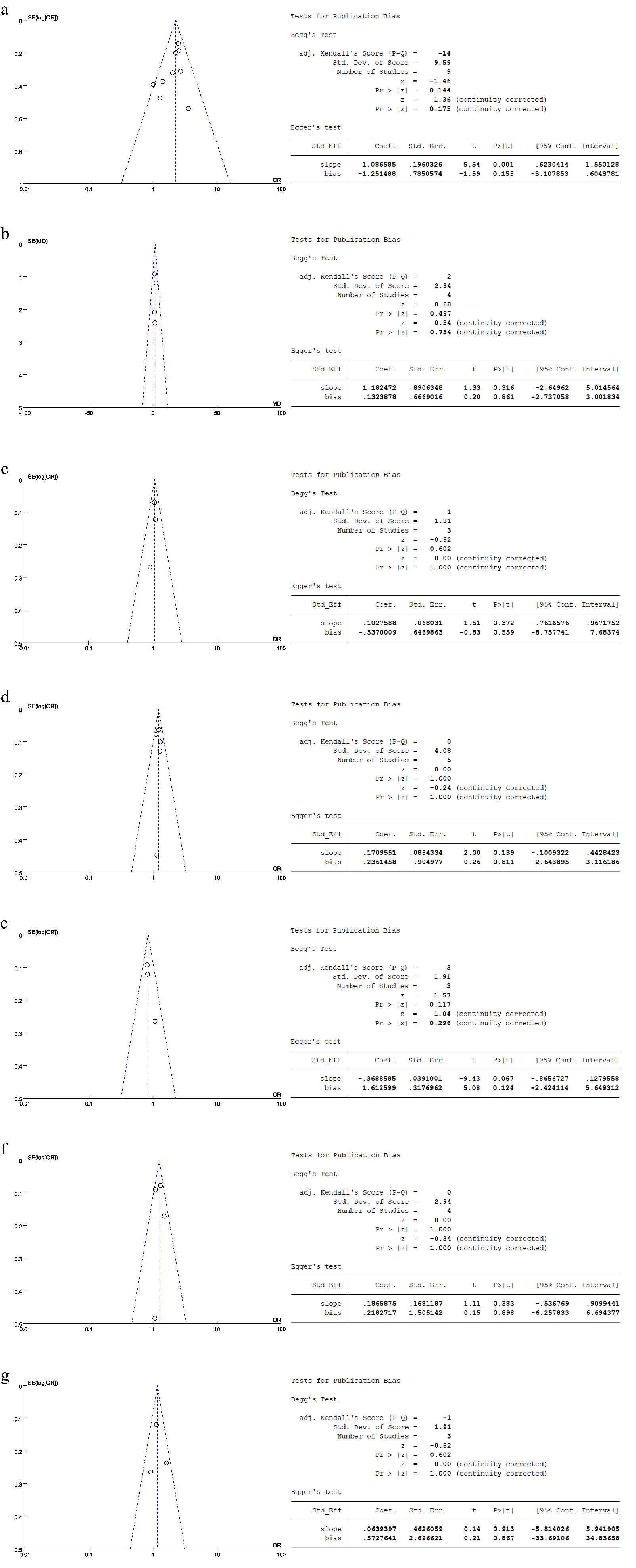


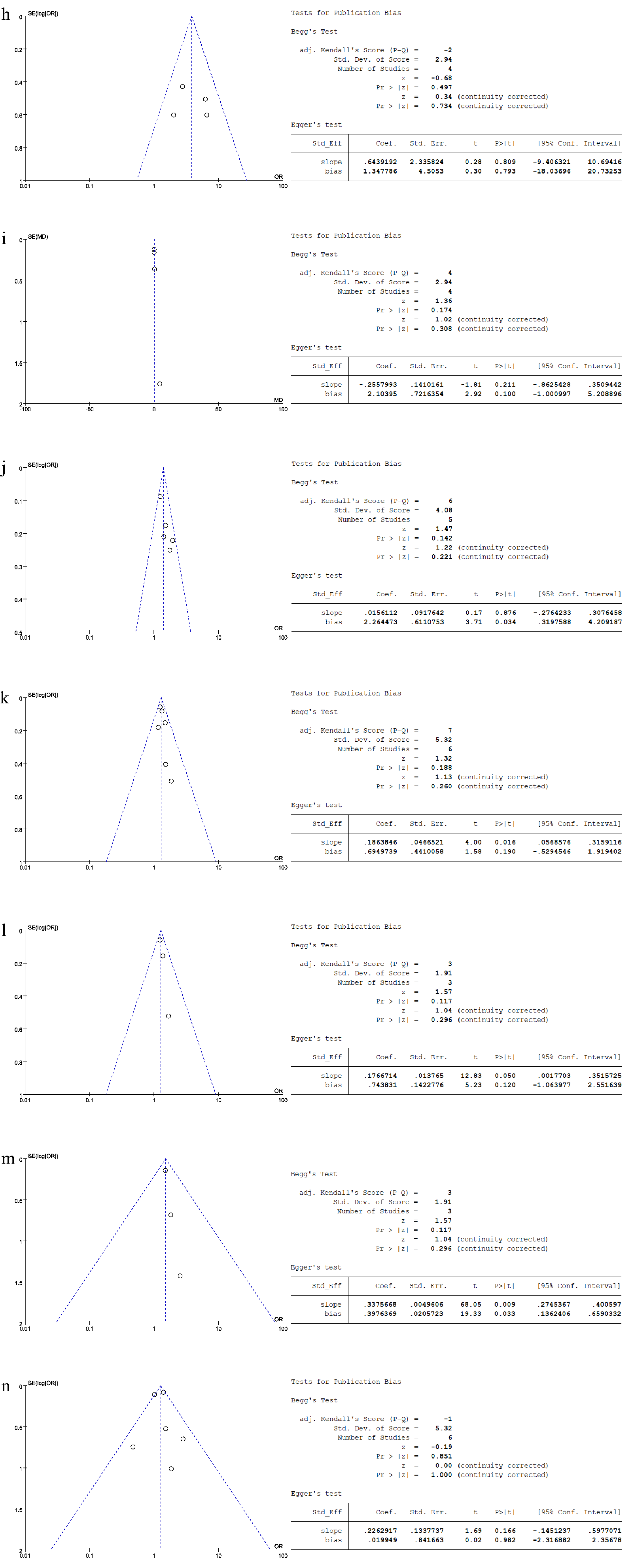


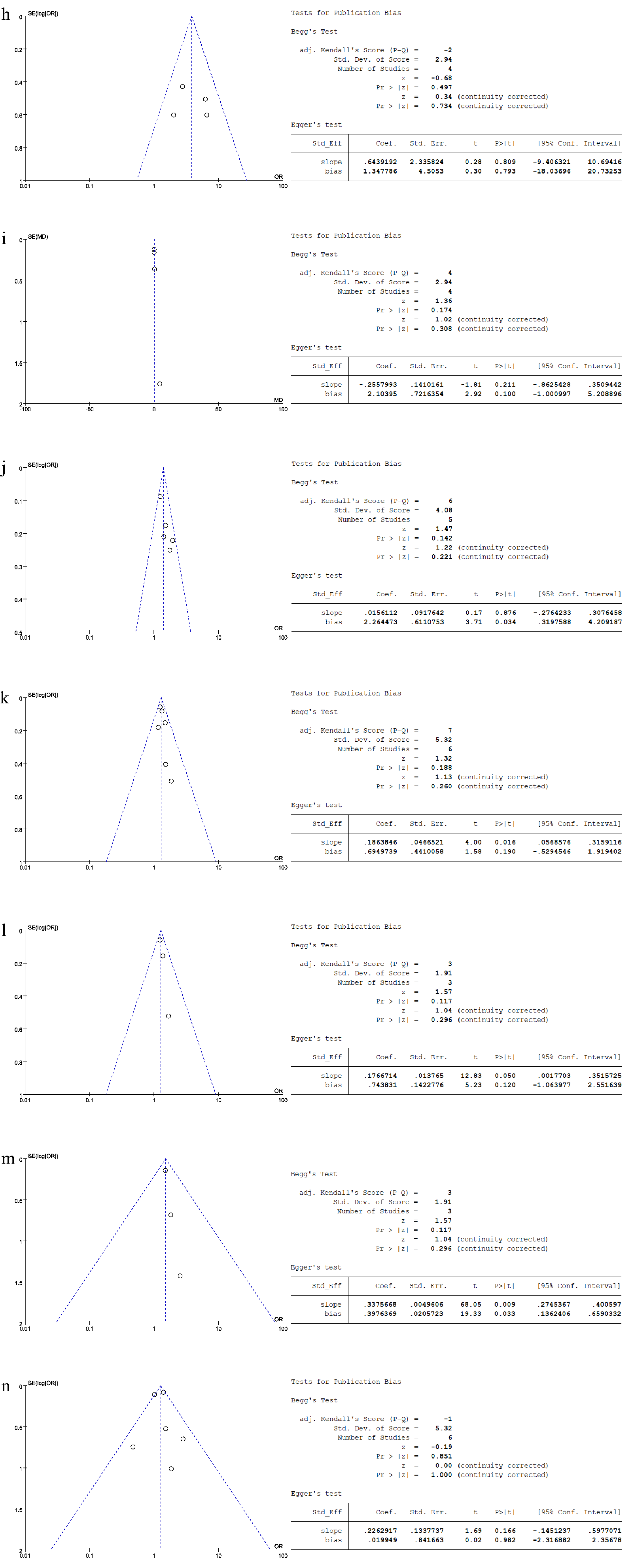


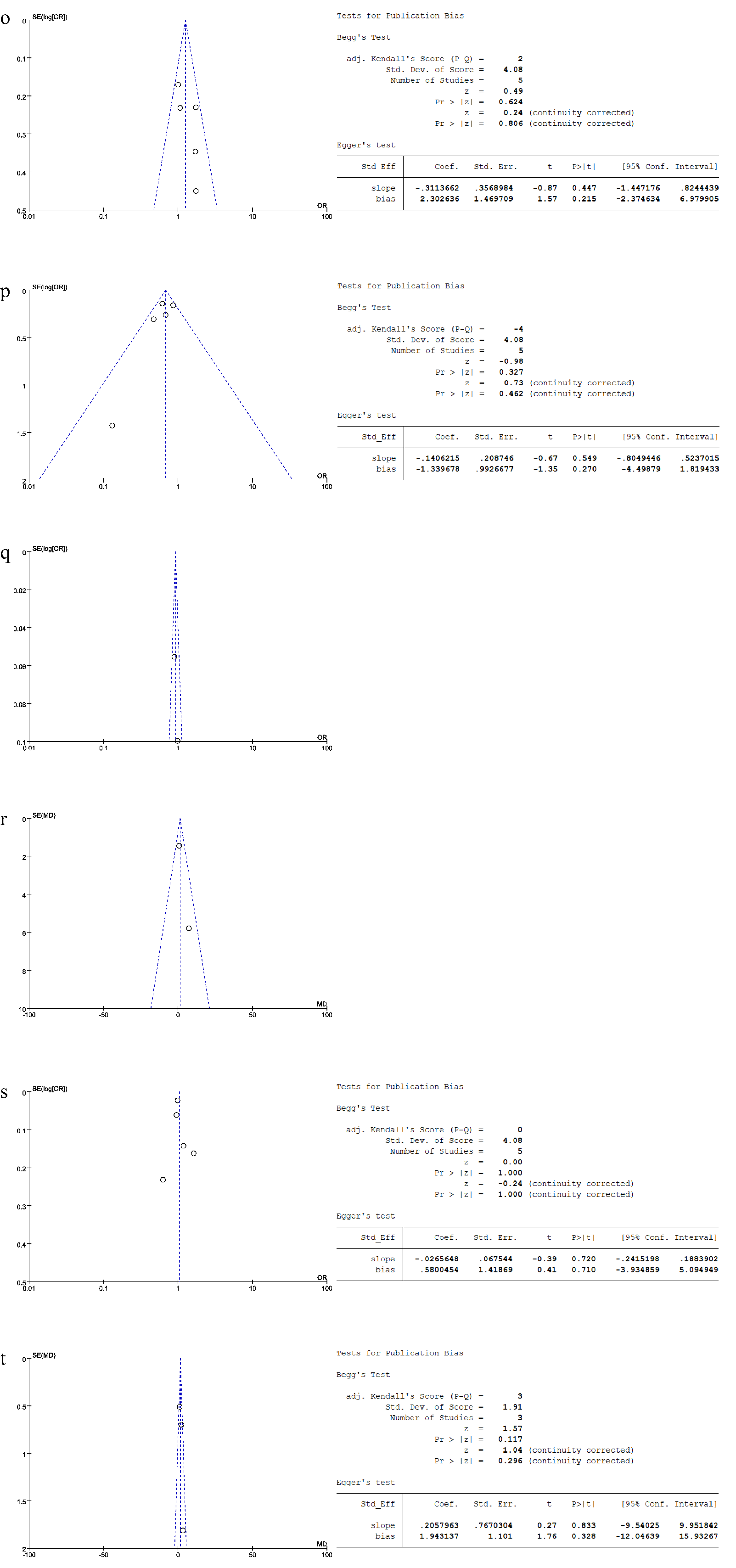


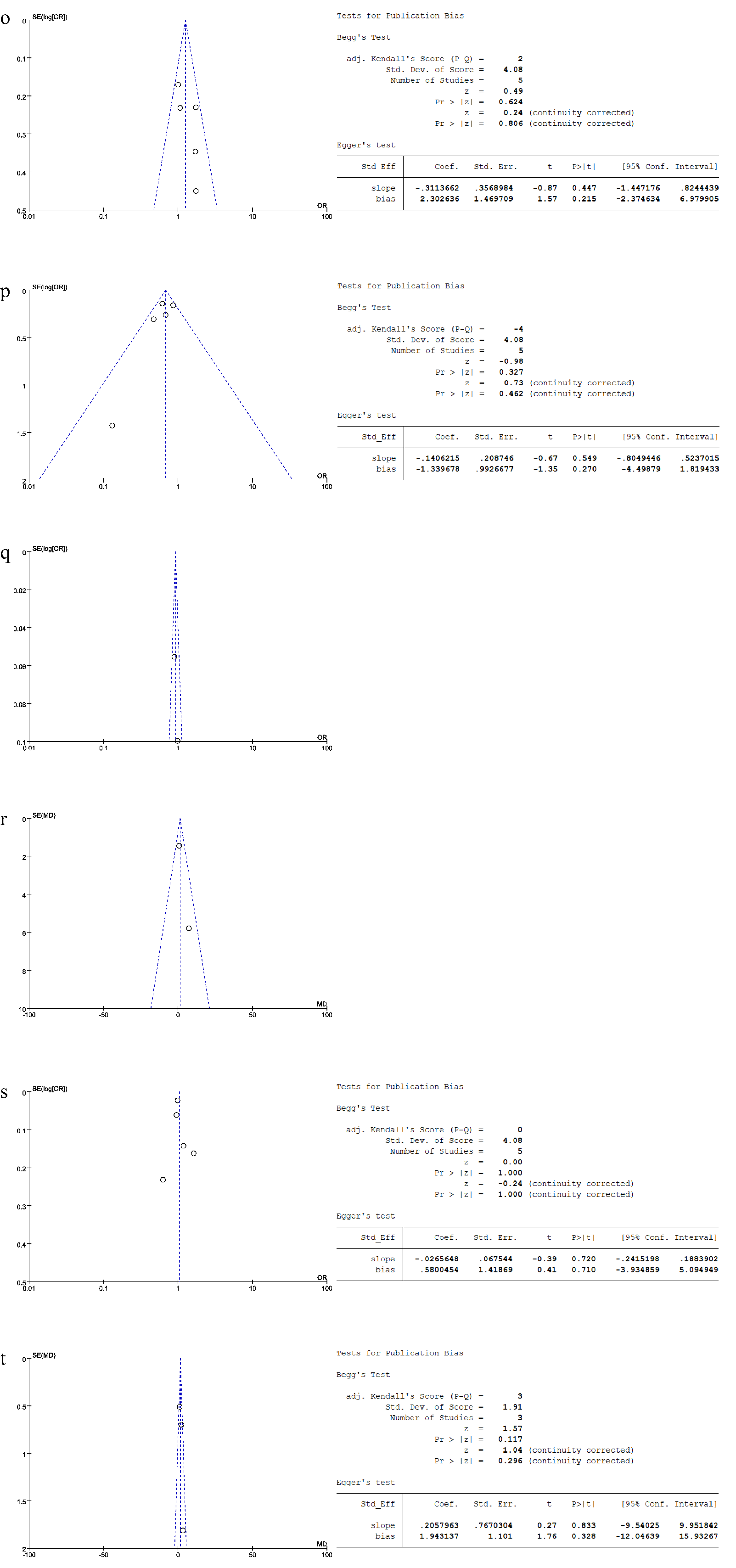


**e-Figure 5** Publication bias (Funnel plot, Begg's test and Egger's test). **a**, Gender; **b**, Age (Continuous); **d**, BMI (≥30.0 kg/m² VS ＜30.0 kg/m²); **e**, BMI (Overweight VS Normal weight); **f**, BMI (Obesity VS Overweight); **g**, BMI (Morbid obesity VS Obesity); **h**, Catheterization; **i**, Total time with urinary catheter; **j**, Blood transfusion; **k**, ASA (≥III VS＜III); **l**, ASA (III VS II); **m**, ASA (II VS I); **n**, Type of anesthesia; **o**, Type of fracture; **p**, Type of surgery; **q**, Operative time; **r**, Time to Surgery (Continuous); **s**, Time to Surgery (＞48h VS ≤48h); **t**, Length of hospital stays.


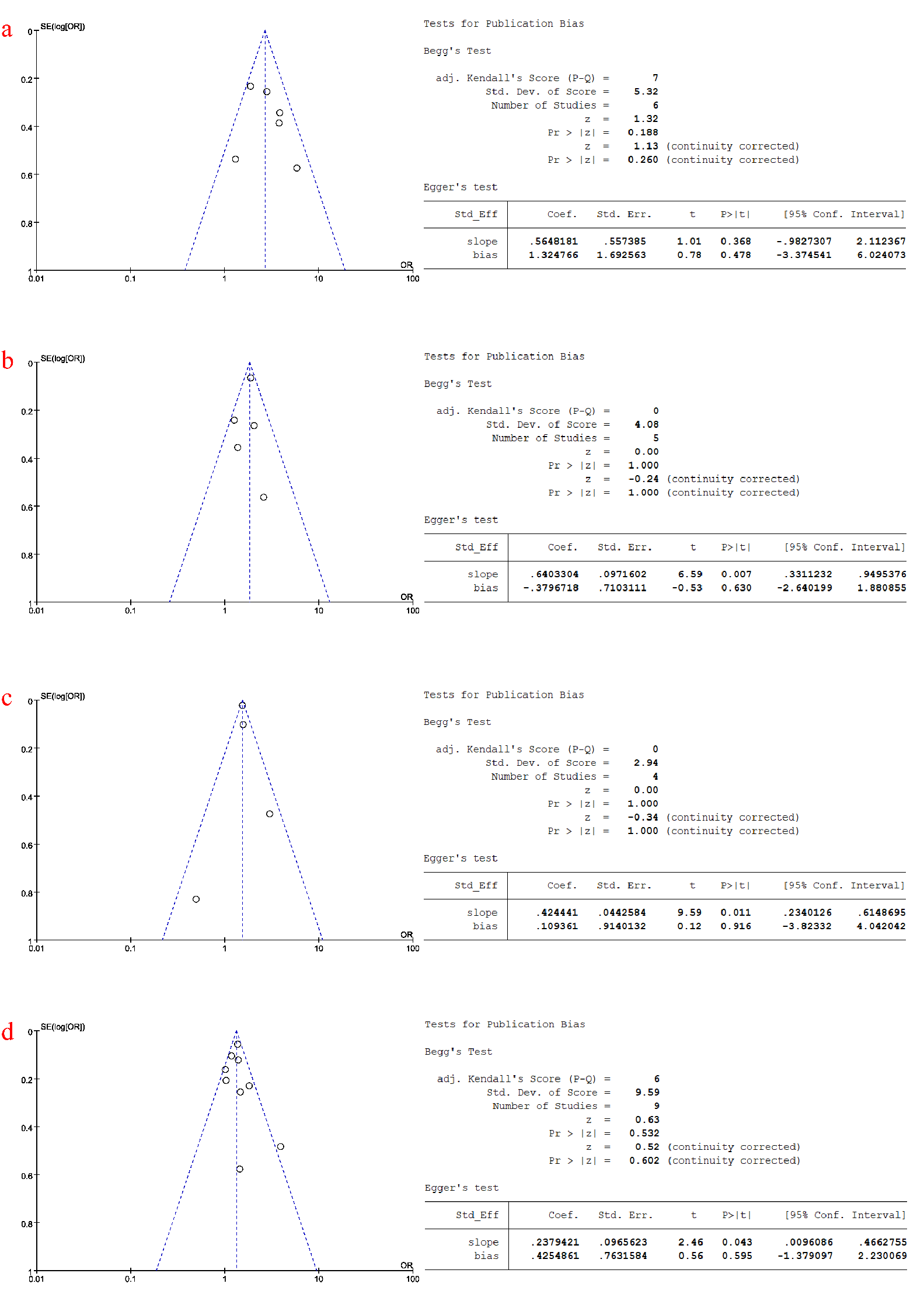


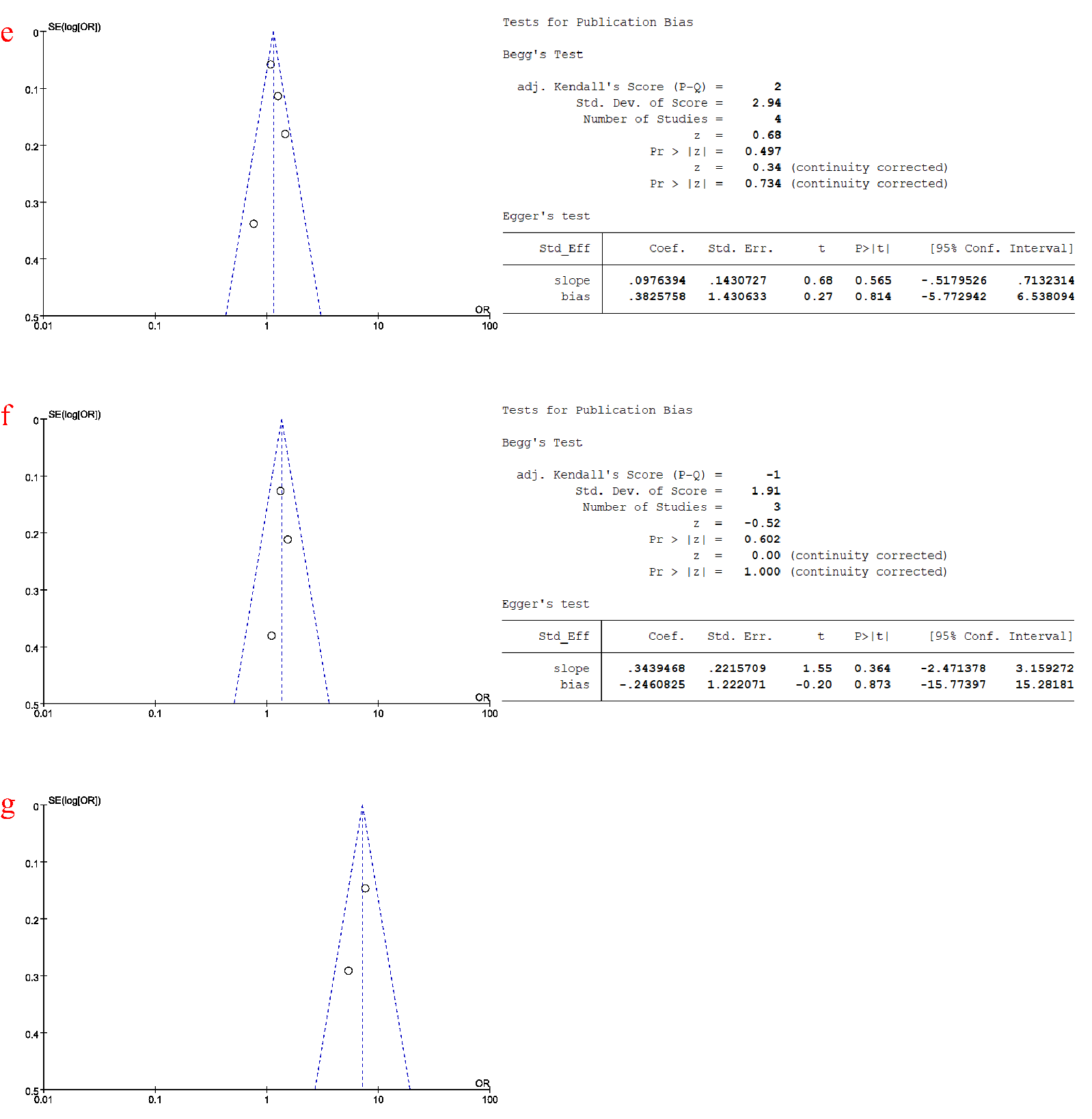


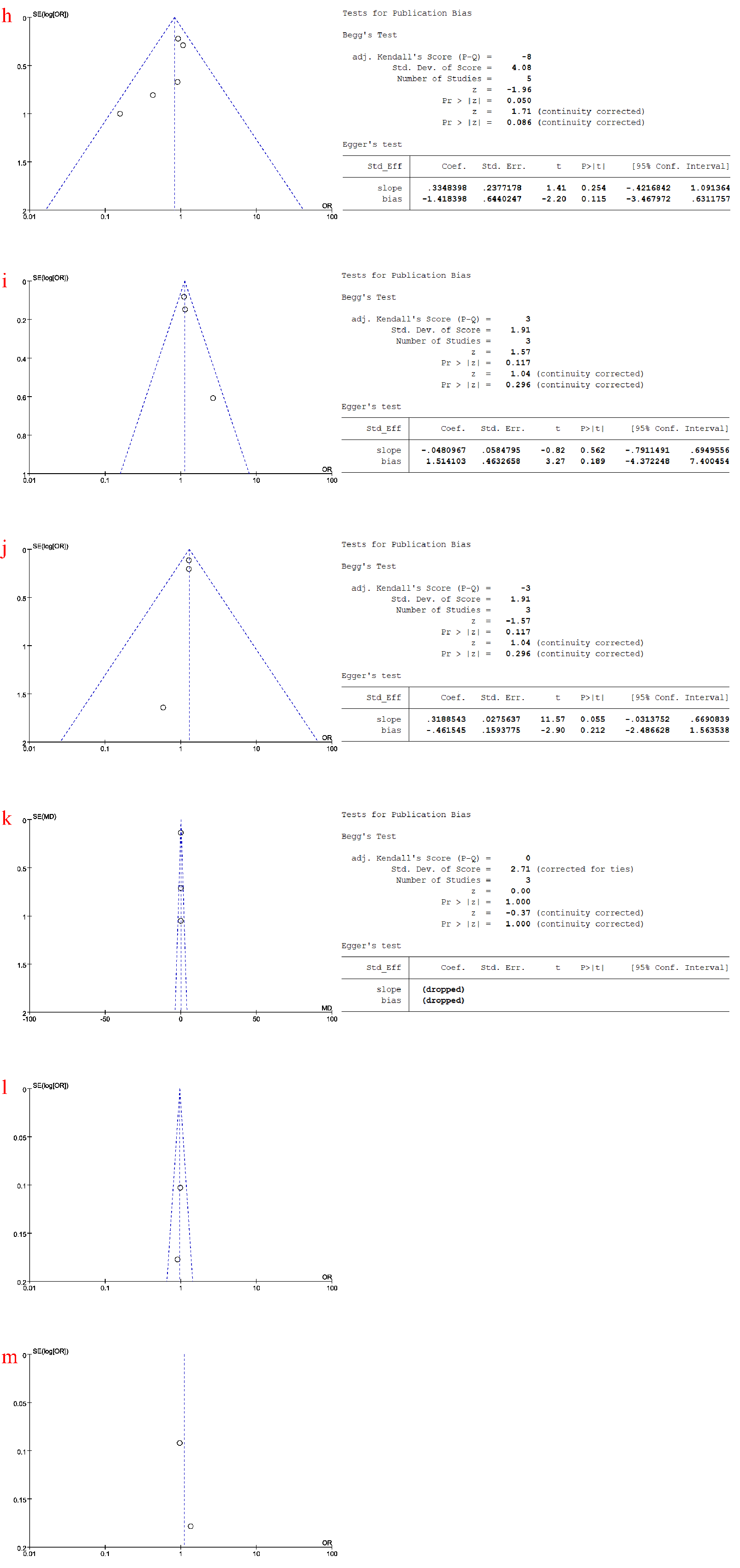


**e-Figure 6** Publication bias (Funnel plot, Begg's test and Egger's test). **a**, Delirium; **b**, Dementia; **c**, Parkinson’s disease; **d**, Diabetes; **e**, Hypertension; **f**, CHF (Congestive Heart Failure); **g**, History of sepsis; **h**, Neoplasm; **i**, COPD (Chronic Obstructive Pulmonary Disease); **j**, Chronic steroid use; **k**, Albumin.
