# Supplementary material for: Risk factors for urinary tract infection in geriatric hip fracture patients: a systematic review and meta-analysis
Source: Front Med (Lausanne). 2024 Feb 9;11:1360058. doi: 10.3389/fmed.2024.1360058 (PMC10884186; doi:10.3389/fmed.2024.1360058)
Supplement: Supplementary file 2 [file Data_Sheet_2.docx]

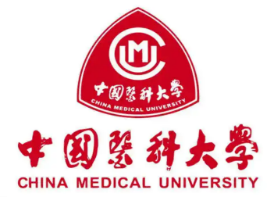
**Highlights**

**The highlights and advantages of our study are outlined below:**

1. To date, numerous systematic reviews have reported on other associated complications of hip fractures, such as pneumonia, delirium, and deep vein thrombosis. However, this study is the only systematic review that focuses specifically on the potential risk factors for urinary tract infection (UTI) in hip fracture patients.
2. This study conducted searches across 5 major databases, including 44 studies, to investigate 24 potential risk factors across 14 different countries. The aim was to provide researchers and clinicians with the most up-to-date and comprehensive information.
3. The heterogeneity among the studies included in this review was relatively low, with the majority employing a fixed-effects model. Each stage of the review process involved independent assessment by two individuals to minimize subjective differences.
4. Our study identified a total of 18 significant risk factors, providing robust evidence and strategies for clinical prediction, guiding early intervention, and implementing targeted management of UTI.
