# Supplementary material for: Risk factors for urinary tract infection in geriatric hip fracture patients: a systematic review and meta-analysis
Source: Front Med (Lausanne). 2024 Feb 9;11:1360058. doi: 10.3389/fmed.2024.1360058 (PMC10884186; doi:10.3389/fmed.2024.1360058)
Supplement: Supplementary file 4 [file Data_Sheet_4.docx]

**Search strategies**

| **PubMed** | ((("Urinary Tract Infections"[Mesh]) OR (((((Infection, Urinary Tract[Title/Abstract]) OR (Infections, Urinary Tract[Title/Abstract])) OR (Tract Infection, Urinary[Title/Abstract])) OR (Tract Infections, Urinary[Title/Abstract])) OR (Urinary Tract Infection[Title/Abstract]))) AND (("Hip Fractures"[Mesh]) OR (((((((((((((((((((((((Femoral Neck Fractures[Title/Abstract]) OR (Femoral Neck Fracture[Title/Abstract])) OR (Femur Neck Fractures[Title/Abstract])) OR (Femur Neck Fracture[Title/Abstract])) OR (Fractures, Hip[Title/Abstract])) OR (Intertrochanteric Fractures[Title/Abstract])) OR (Fractures, Intertrochanteric[Title/Abstract])) OR (Trochanteric Fractures[Title/Abstract])) OR (Fractures, Trochanteric[Title/Abstract])) OR (Trochlear Fractures, Femur[Title/Abstract])) OR (Femur Trochlear Fracture[Title/Abstract])) OR (Femur Trochlear Fractures[Title/Abstract])) OR (Fracture, Femur Trochlear[Title/Abstract])) OR (Fractures, Femur Trochlear[Title/Abstract])) OR (Trochlear Fracture, Femur[Title/Abstract])) OR (Femoral Trochlear Fractures[Title/Abstract])) OR (Femoral Trochlear Fracture[Title/Abstract])) OR (Fracture, Femoral Trochlear[Title/Abstract])) OR (Fractures, Femoral Trochlear[Title/Abstract])) OR (Trochlear Fracture, Femoral[Title/Abstract])) OR (Trochlear Fractures, Femoral[Title/Abstract])) OR (Subtrochanteric Fractures[Title/Abstract])) OR (Fractures, Subtrochanteric[Title/Abstract])))) AND ((("Risk"[Mesh])) OR (((((((((((((((((((((((Risks[Title/Abstract]) OR (Relative Risk[Title/Abstract])) OR (Relative Risks[Title/Abstract])) OR (Risk, Relative[Title/Abstract])) OR (Risks, Relative[Title/Abstract])) OR (Risk Factors[Title/Abstract])) OR (Factor, Risk[Title/Abstract])) OR (Risk Factor[Title/Abstract])) OR (Influencing factors[Title/Abstract])) OR (relevant factors[Title/Abstract])) OR (Influencing factor[Title/Abstract])) OR (relevant factor[Title/Abstract])) OR (predictor[Title/Abstract])) OR (associated factor[Title/Abstract])) OR (associated factors[Title/Abstract])) OR (dangerous factor[Title/Abstract])) OR (dangerous factors[Title/Abstract])) OR (related factor[Title/Abstract])) OR (related factors[Title/Abstract])) OR (influential factor[Title/Abstract])) OR (influential factors[Title/Abstract])) OR (Protective Factor[Title/Abstract])) OR (Protective Factors[Title/Abstract]))) |
| --- | --- |
| **Embase** | ('urinary tract infection'/exp OR 'infection, urinary tract':ab,ti OR 'infections, urinary tract':ab,ti OR 'tract infection, urinary':ab,ti OR 'tract infections, urinary':ab,ti OR 'urinary tract infections':ab,ti) AND ('hip fracture'/exp OR 'hip fractures':ab,ti OR 'femoral neck fractures':ab,ti OR 'femoral neck fracture':ab,ti OR 'femur neck fractures':ab,ti OR 'femur neck fracture':ab,ti OR 'fractures, hip':ab,ti OR 'intertrochanteric fractures':ab,ti OR 'fractures, intertrochanteric':ab,ti OR 'trochanteric fractures':ab,ti OR 'fractures, trochanteric':ab,ti OR 'trochlear fractures, femur':ab,ti OR 'femur trochlear fracture':ab,ti OR 'femur trochlear fractures':ab,ti OR 'fracture, femur trochlear':ab,ti OR 'fractures, femur trochlear':ab,ti OR 'trochlear fracture, femur':ab,ti OR 'femoral trochlear fractures':ab,ti OR 'femoral trochlear fracture':ab,ti OR 'fracture, femoral trochlear':ab,ti OR 'fractures, femoral trochlear':ab,ti OR 'trochlear fracture, femoral':ab,ti OR 'trochlear fractures, femoral':ab,ti OR 'subtrochanteric fractures':ab,ti OR 'fractures, subtrochanteric':ab,ti) AND ('risk'/exp AND 'risks':ab,ti OR 'relative risk':ab,ti OR 'relative risks':ab,ti OR 'risk, relative':ab,ti OR 'risks, relative':ab,ti OR 'risk factors':ab,ti OR 'factor, risk':ab,ti OR 'risk factor':ab,ti OR 'influencing factors':ab,ti OR 'relevant factors':ab,ti OR 'influencing factor':ab,ti OR 'relevant factor':ab,ti OR 'predictor':ab,ti OR 'associated factor':ab,ti OR 'associated factors':ab,ti OR 'dangerous factor':ab,ti OR 'dangerous factors':ab,ti OR 'related factor':ab,ti OR 'related factors':ab,ti OR 'influential factor':ab,ti OR 'influential factors':ab,ti OR 'Protective Factor':ab,ti OR 'Protective Factors':ab,ti) |
| **Cochrane Library** | #1 (Urinary Tract Infections):ab,ti,kw OR (Infection, Urinary Tract):ab,ti,kw OR (Infections, Urinary Tract):ab,ti,kw OR (Tract Infection, Urinary):ab,ti,kw OR (Tract Infections, Urinary):ab,ti,kw OR (Urinary Tract Infection):ab,ti,kw  #2 (Hip Fractures):ab,ti,kw OR (Femoral Neck Fractures):ab,ti,kw OR (Femoral Neck Fracture):ab,ti,kw OR (Femur Neck Fractures):ab,ti,kw OR (Femur Neck Fracture):ab,ti,kw OR (Fractures, Hip):ab,ti,kw OR (Intertrochanteric Fractures):ab,ti,kw OR (Fractures, Intertrochanteric):ab,ti,kw OR (Trochanteric Fractures):ab,ti,kw OR (Fractures, Trochanteric):ab,ti,kw OR (Trochlear Fractures, Femur):ab,ti,kw OR (Femur Trochlear Fracture):ab,ti,kw OR (Femur Trochlear Fractures):ab,ti,kw OR (Fracture, Femur Trochlear):ab,ti,kw OR (Fractures, Femur Trochlear):ab,ti,kw OR (Trochlear Fracture, Femur):ab,ti,kw OR (Femoral Trochlear Fractures):ab,ti,kw OR (Femoral Trochlear Fracture):ab,ti,kw OR (Fracture, Femoral Trochlear):ab,ti,kw OR (Fractures, Femoral Trochlear):ab,ti,kw OR (Trochlear Fracture, Femoral):ab,ti,kw OR (Trochlear Fractures, Femoral):ab,ti,kw OR (Subtrochanteric Fractures):ab,ti,kw OR (Fractures, Subtrochanteric):ab,ti,kw  #3 (Risk):ab,ti,kw OR (Risks):ab,ti,kw OR (Relative Risk):ab,ti,kw OR (Relative Risks):ab,ti,kw OR (Risk, Relative):ab,ti,kw OR (Risks, Relative):ab,ti,kw OR (Risk Factors):ab,ti,kw OR (Factor, Risk):ab,ti,kw OR (Risk Factor):ab,ti,kw OR (Influencing factors):ab,ti,kw OR (relevant factors):ab,ti,kw OR (Influencing factor):ab,ti,kw OR (relevant factor):ab,ti,kw OR (predictor):ab,ti,kw OR (associated factor):ab,ti,kw OR (associated factors):ab,ti,kw OR (dangerous factor):ab,ti,kw OR (dangerous factors):ab,ti,kw OR (related factor):ab,ti,kw OR (related factors):ab,ti,kw OR (influential factor):ab,ti,kw OR (influential factors):ab,ti,kw OR (Protective Factor):ab,ti,kw OR (Protective Factors):ab,ti,kw  #4 #1 AND #2 AND #3 46 |
| **Web of Science** | 1: TS=(Urinary Tract Infections OR Infection, Urinary Tract OR Infections, Urinary Tract OR Tract Infection, Urinary OR Urinary Tract Infection OR Tract Infections, Urinary)  2: TS=(Hip Fractures OR Femoral Neck Fractures OR Femoral Neck Fracture OR Femur Neck Fractures OR Fractures, Hip OR Femur Neck Fracture OR Intertrochanteric Fractures OR Fractures, Intertrochanteric OR Trochanteric Fractures OR Fractures, Trochanteric OR Trochlear Fractures, Femur OR Femur Trochlear Fracture OR Femur Trochlear Fractures OR Fracture, Femur Trochlear OR Fractures, Femur Trochlear OR Trochlear Fracture, Femur OR Femoral Trochlear Fractures OR Femoral Trochlear Fracture OR Fracture, Femoral Trochlear OR Fractures, Femoral Trochlear OR Trochlear Fracture, Femoral OR Trochlear Fractures, Femoral OR Subtrochanteric Fractures OR Fractures, Subtrochanteric)  3: TS=(Risk OR Risks OR Relative Risk OR Relative Risks OR Risks, Relative OR Risk, Relative OR Risk Factors OR Factor, Risk OR Risk Factor OR Influencing factors OR relevant factors OR Influencing factor OR relevant factor OR predictor OR associated factor OR associated factors OR dangerous factor OR dangerous factors OR related factor OR related factors OR influential factor OR influential factors OR Protective Factor OR Protective Factors)  4: #3 AND #2 AND #1 |
| **Scopus** | TITLE-ABS-KEY ("Urinary Tract Infections" OR "Infection, Urinary Tract" OR "Infections, Urinary Tract" OR "Tract Infection, Urinary" OR "Urinary Tract Infection" OR "Tract Infections, Urinary") AND TITLE-ABS-KEY ("Hip Fractures" OR "Femoral Neck Fractures" OR "Femoral Neck Fracture" OR "Femur Neck Fractures" OR "Fractures, Hip" OR "Femur Neck Fracture" OR "Intertrochanteric Fractures" OR "Fractures, Intertrochanteric" OR "Trochanteric Fractures" OR "Fractures, Trochanteric" OR "Trochlear Fractures, Femur" OR "Femur Trochlear Fracture" OR "Femur Trochlear Fractures" OR "Fracture, Femur Trochlear" OR "Fractures, Femur Trochlear" OR "Trochlear Fracture, Femur" OR "Femoral Trochlear Fractures" OR "Femoral Trochlear Fracture" OR "Fracture, Femoral Trochlear" OR "Fractures, Femoral Trochlear" OR "Trochlear Fracture, Femoral" OR "Trochlear Fractures, Femoral" OR "Subtrochanteric Fractures" OR "Fractures, Subtrochanteric") AND TITLE-ABS-KEY ("Risk" OR "Risks" OR "Relative Risk" OR "Relative Risks" OR "Risks, Relative" OR "Risk, Relative" OR "Risk Factors" OR "Factor, Risk" OR "Risk Factor" OR "Influencing factors" OR "relevant factors" OR "Influencing factor" OR "relevant factor" OR "predictor" OR "associated factor" OR "associated factors" OR "dangerous factor" OR "dangerous factors" OR "related factor" OR "related factors" OR "influential factor" OR "influential factors" OR "Protective Factor" OR "Protective Factors") |
